# The Genetic Basis of Childhood Obesity: A Systematic Review

**DOI:** 10.3390/nu15061416

**Published:** 2023-03-15

**Authors:** Aikaterini Vourdoumpa, George Paltoglou, Evangelia Charmandari

**Affiliations:** 1 Division of Endocrinology, Metabolism and Diabetes, First Department of Pediatrics, National and Kapodistrian University of Athens Medical School, ‘Aghia Sophia’ Children’s Hospital, 11527 Athens, Greece; 2 Division of Endocrinology and Metabolism, Center for Clinical, Experimental Surgery and Translational Research, Biomedical Research Foundation of the Academy of Athens, 11527 Athens, Greece

**Keywords:** SNPs, CNVs, obesity, BMI, body composition, lifestyle intervention, children, adolescents

## Abstract

Overweight and obesity in childhood and adolescence represents one of the most challenging public health problems of our century owing to its epidemic proportions and the associated significant morbidity, mortality, and increase in public health costs. The pathogenesis of polygenic obesity is multifactorial and is due to the interaction among genetic, epigenetic, and environmental factors. More than 1100 independent genetic loci associated with obesity traits have been currently identified, and there is great interest in the decoding of their biological functions and the gene–environment interaction. The present study aimed to systematically review the scientific evidence and to explore the relation of single-nucleotide polymorphisms (SNPs) and copy number variants (CNVs) with changes in body mass index (BMI) and other measures of body composition in children and adolescents with obesity, as well as their response to lifestyle interventions. Twenty-seven studies were included in the qualitative synthesis, which consisted of 7928 overweight/obese children and adolescents at different stages of pubertal development who underwent multidisciplinary management. The effect of polymorphisms in 92 different genes was assessed and revealed SNPs in 24 genetic loci significantly associated with BMI and/or body composition change, which contribute to the complex metabolic imbalance of obesity, including the regulation of appetite and energy balance, the homeostasis of glucose, lipid, and adipose tissue, as well as their interactions. The decoding of the genetic and molecular/cellular pathophysiology of obesity and the gene–environment interactions, alongside with the individual genotype, will enable us to design targeted and personalized preventive and management interventions for obesity early in life.

## 1. Introduction

Overweight and obesity have reached epidemic proportions in contemporary societies, not only in adulthood but also in childhood and adolescence [1]. The prevalence of overweight and obesity in the pediatric population has more than quadrupled over the last 40 years, ranging from 4% in 1975 to >18% in 2016 [1]. Children with obesity demonstrate a 5-fold increased risk to remain obese in adulthood [2], especially in cases of severe obesity and/or obesity in one or both parents [3]. In addition, data from the World Health Organization (WHO) European Region estimate that over 60% of prepubertal children with overweight will be overweight as young adults [4]. According to the International Classification of Diseases 11 (ICD-11), obesity is a chronic complex disease, characterized by excess body fat accumulation, which adversely affects human health [5]. Increased adiposity in childhood results in numerous comorbidities, including obstructive sleep apnea, hypertension, left ventricular hypertrophy, acanthosis nigricans, insulin resistance and diabetes mellitus type 2, nonalcoholic fatty liver disease (NAFLD), polycystic ovary syndrome (PCOS), orthopedic problems, and impaired psychological health, which may present as early as childhood or adolescence [3,6]. Moreover, childhood obesity has been independently correlated with cardiovascular and metabolic diseases, cancer, and early mortality later in adult life [7,8,9,10,11,12].

The pathogenesis of polygenic obesity, the most common form of obesity, is multifactorial with genetic, epigenetic, and environmental factors all interacting among them and contributing to its development [13,14,15,16]. The term “obesogenic environment” refers to the role that environmental factors may play in determining both nutrition and physical activity and how they result in obesity [17]. In principle, weight gain occurs when energy intake (calories consumed) exceeds total daily energy expenditure for a prolonged period of time [15]. A current pathogenetic model states that the obesogenic environment, on one hand, triggers obesity-predisposing genes, and on the other hand, results in epigenetic changes, which both consequently contribute to the development of obesity [14,18,19]. Studies in twins show that the genetic heritability of obesity is as high as 47–90% [20], and equally high rates of inheritance have been associated with robust measures of adiposity, such as increased waist-to-hip ratio and others [21,22]. The inheritance pattern of polygenic obesity does not follow the principles of Mendelian inheritance but is the result of common genetic variations following the inheritance model of other complex diseases [23]. 

Genetic variations include single-nucleotide polymorphisms (SNPs), copy number variants (CNVs), and small insertions and deletions [15,23], each contributing to the development of obesity according to either the common-disease–common-variant hypothesis (common complex traits are largely due to common variants with small-to-modest effect) or the opposing, rare-variant hypothesis (common complex traits are the summation of low-frequency, high-penetrance variants) [23,24]. The upper percentiles of body mass index (BMI; the weight in kilograms divided by the square of the height in meters) distribution have been associated with rarer variants of high penetrance, explaining a proportion of the heritability [15]. In addition, the influence of the genotype in the pathogenesis of obesity follows a biphasic model, increasing with increasing age in childhood and adolescence, and attenuating with increasing age in adulthood [20]. More than 1100 independent loci associated with obesity have been identified to date through genome-wide association studies (GWAS) [23]. Although there is an overlap between variants related to obesity in childhood, adolescence, and adulthood, unique variants have also been associated with obesity in childhood [25,26,27]. Some of the genetic loci associated with the pathogenesis of obesity are *MC4R*, *BDNF*, *SH2B1*, *POMC*, *LEP*, *LEPR*, *NPY*, *SIM1*, *NTRK2*, and *PCSK1* [15,23], as well as CNVs in 11q11 (*OR4P4*, *OR4S2*, *OR4C6*), 1p21.1 (*AΜΥ1*), 10q11.22 (*NPY4R*), 10q26.3 (*CYP2E1*), 16q12.2 (*FTO*), 16p12.3 (*GPRC5b*), and 4q25 loci [28]. The most recent Genetic Investigation of ANthropometric Traits (GIANT) meta-analysis of 700,000 people featured 941 nearly independent SNPs associated with BMI, with the lowest minor allele frequency (MAF) being 1.6% and the minimum per allele effect being 0.04 kg/m^2^ per allele [23]. However, the variation of BMI currently explained by GWAS is limited to 6% [29]. Therefore, it is of great interest to decode the biological functions of the obesity-associated genetic loci identified through GWAS in order to enrich our understanding of the pathogenesis of obesity [23].

The obese state is characterized by a complex metabolic imbalance, associated with major changes in metabolic processes, including the central and peripheral nervous system regulation of energy balance, glucose, lipid, and adipose tissue homeostasis, as well as their interactions [30]. It is well-established that the central nervous system (CNS) plays a fundamental role in the control of food intake and energy homeostasis through genes expressed in the hypothalamus and pituitary gland, as well as in other brain regions involved in learning, cognition, addiction, reward, and emotional responses, including the hippocampus, the limbic system, the insula, and substantia nigra [23]. Among those, the leptin–melanocortin pathway and neuronal circuits involved in structural organization and plasticity, as well as their downstream signaling pathways, such as the dopaminergic and serotonergic pathways, mediate appetite and energy balance [23,31,32].

Furthermore, in the obese state, multiple changes in adipocyte-related gene expression modulate both biological pathways and the hormonal milieu of the organism by altering the expression of adipokines [33]. In addition, adipogenesis is characterized by adipose tissue hyperplasia (increased cell number), which appears mainly in the early stages of adipose tissue development, and hypertrophy (increased cell size), which occurs prior to hyperplasia to meet the need for additional fat storage capacity in the progression of obesity [34]. However, it has proven difficult to understand how energy balance and genetics specifically and differentially affect hyperplasia or hypertrophy of adipocytes that contribute to the metabolic state of the organism in obesity [35]. In metabolically healthy obesity, new, small adipocytes are formed (hyperplasia), while in impaired adipogenesis, the existing adipocytes become hypertrophic, which is a state associated with metabolic disorders of obesity favoring stress-induced hypoxia, low-grade inflammation, and insulin resistance [35]. Further complexity is added when considering the different histological forms of adipocytes (white, brown, and beige) [36,37].

A cross-talk between adipose tissue and the immune system takes place, with multiple genes associated with obesity encoding immune-cell-related proteins [38]. An excess in macronutrients intake leads to obesity and creates a chronic low-grade aseptic systemic inflammation [39]. Indeed, enlargement of adipocytes creates hypoxic conditions, enhancing inflammatory signaling, adipose tissue fibrosis, cellular death, and macrophage infiltration, with a shift from an anti-inflammatory M2-like to a pro-inflammatory M1-like phenotype, thereby increasing inflammatory mediators, such as IL-6 and TNF-α [39]. The proinflammatory state of obesity is also characterized by an anti-inflammatory adaptive response, characterized by changes in T-cell subpopulations in the adipose tissue, such as a CD8+ T cell activation, a decrease in Tregs and Th2 cells, and an increase in Th1 and Th17 cells, as well as disruption of the expression of many adipokines, including adiponectin, resistin, and visfatin [39]. Finally, microbial agents, such as viruses and viral-like agents, also play an important role in the pathogenesis of obesity, a phenomenon referred to as “infectobesity” [40].

To the best of our knowledge, very few studies have reviewed the gene–environment interactions in relation to BMI and changes in body composition after the implementation of lifestyle interventions to combat obesity, and they either focused on adult populations [41,42] or examined variations in single genes [43,44,45]. The aim of the present study was to systematically review the literature in order to explore the relation of SNPs and CNVs with changes in BMI and other measures of body composition in children and adolescents with overweight or obesity who undertook a lifestyle intervention program.

## 2. Materials and Methods

### 2.1. Study Design

The present study was designed and conducted in accordance with the Preferred Reporting Items for Systematic Reviews and Meta-Analyses (PRISMA) protocol [46]. The review was registered in the International Prospective Register of Ongoing Systematic Reviews (PROSPERO Registration Number: CRD42022313595). To formulate the objectives of our review, the PICO (P—Populations/People/Patient/Problem, I—Intervention(s), C—Comparison, O—Outcome) model was implemented (Table 1).

### 2.2. Eligibility Criteria

The present review included interventional cohorts and control trials, which examined the change in ΒΜΙ and/or body composition in relation to the genotype (SNVs and/or CNVs) in children and adolescents with overweight or obesity who followed a lifestyle intervention management program. Reviews, editorials, books and book chapters, notes, letters, conference papers, and surveys were not eligible for inclusion. Moreover, there were no restrictions on language, year of publication, or geographic location in order to ensure a broad and comprehensive literature search and minimize bias. Inclusion and exclusion criteria are presented in Table 2.

### 2.3. Literature Search

We conducted a literature search in three databases, Medline (PubMed), Scopus, and Cochrane Central Register of Controlled Trials (CENTRAL), prior to February 2022. Designing the search strategy, five main term categories were identified and combined using “AND”: (1) SNVs or CNVs, (2) BMI and body composition measures, (3) children and adolescents, (4) obesity, and (5) lifestyle interventions. Synonyms and representative key words of the terms within each of the five categories were defined by the Medical Subject Headings (MeSH) database and by a literature review [47], and they were combined using “OR”.

The final search in Medline was formulated as follows: ((((((((allel*) OR (variant*)) OR (polymorphi*)) OR (CNV)) OR (copy number variation*)) AND (((((((((((((((((((((BMI) OR (“mass”)) OR (fat)) OR (adipos*)) OR (circumference*)) OR (skinfold*)) OR (waist*)) OR (body* composition*)) OR (plethysmograph*)) OR (hydrodensitometr*)) OR (hydrometry)) OR (isotope* dilution*)) OR (bioimpedance)) OR (impedance)) OR (Dual-energy X-ray*)) OR (DXA)) OR (DEXA)) OR (MRI)) OR (Magnetic resonance)) OR (Compute* tomograph*)) OR (“CT”))) AND (((child*) OR (pediatric*)) OR (adolescen*))) AND ((obes*) OR (overweight))) AND (((((((intervention*) OR (diet*)) OR (nutrition*)) OR (exercis*)) OR (“physical activit*”)) OR (fitness*)) OR (weight loss)). This search strategy was appropriately adjusted to match the rest of the databases.

Furthermore, an additional backward-and-forward search for identification of eligible studies was performed in the reference articles of the included studies.

### 2.4. Study Selection

The identification, screening, eligibility, and inclusion process were performed by two independent reviewers (A.V. and G.P.), who remained blinded to each other’s work. Duplicates were removed using the EndNote X7 software. The eligibility assessment of the studies for meeting inclusion and exclusion criteria was made in two stages. The first eligibility check was based on the title and abstract, while the second eligibility check evaluated the full articles’ text. Discussion between the authors was used to resolve any data conflicts that occurred.

### 2.5. Data Extraction, Outcomes and Data Synthesis

Two authors (A.V. and G.P.), working independently, extracted the data on standardized Excel work templates, and any disagreements were solved through discussion, achieving a consensus. The extracted data items concerned the following fields: general study information (study type, outcomes examined, sample size, country, age, sex, pubertal status, obesity definition, study parameters examined, main findings) (Table 3), obesity intervention characteristics (type of intervention, dietary, exercise and behavioral intervention description, compliance measurement, dropout rate) (available in Supplementary Table S1). and genotype-related information (genes, SNPs, risk alleles, alleles combinations and GRS examined, effect of genotype after intervention on weight, ΒΜΙ/ΒΜΙ-SDS, body composition, genetic inheritance model) (available in Supplementary Table S4).

The main outcomes examined in the present systematic review were genotype-related changes of BMI/BMI-SDS and/or body composition after lifestyle interventions implemented as part of the management of overweight and obesity. Secondary, we evaluated the specific components of the interventions, leading to significant changes, as well as the effect of the rest of the extracted data mentioned above. A qualitive synthesis of the data took place based on these outcomes.

### 2.6. Validity Assessment

Further to the above, the same two authors (A.V. and G.P.) separately assessed quality using the Newcastle–Ottawa Scale (NOS) [75] for cohort studies and randomized control trials. Any mismatches between the reviewers were discussed to reach a consensus.

The three main domains of NOS and the individual subgroups within them were evaluated, as shown in Supplementary Tables S2 and S3, through a star-awarding system attributed to each subsection. Studies were categorized as being of good, fair, or poor quality. To assess the adequate duration of the follow-up period for the outcome to occur in the respective subsection of the Exposure category in NOS for cohort studies, a threshold of 6 months for the lifestyle interventions was applied [76].

## 3. Results

**Figure 1:**
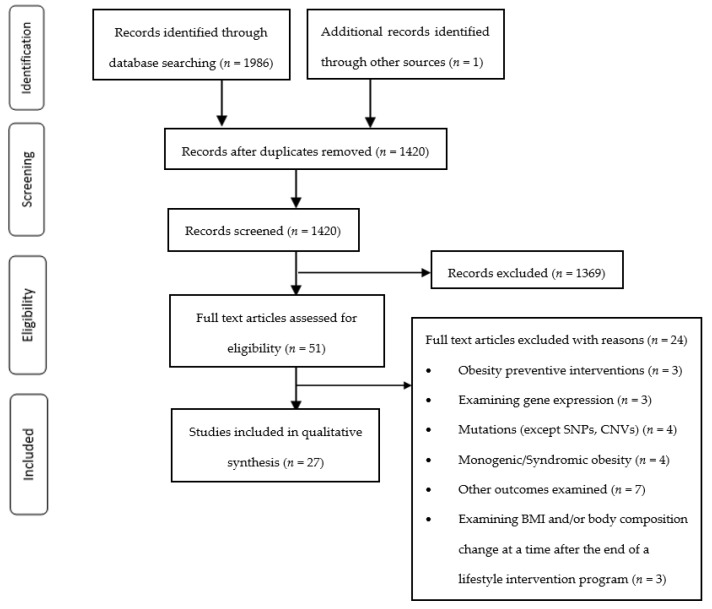
Flow diagram describing the identification, screening, eligibility, and inclusion process.

Figure 1 summarizes the identification, screening, eligibility, and inclusion process of articles for review. Our initial database search identified 1986 articles, while one additional article was discovered through backward-and-forward reference searches. The articles were uploaded in EndNote X7, and after the removal of duplicates, 1420 studies remained for further evaluation. The screening of titles and abstracts resulted in the removal of 1369 studies due to their not meeting the inclusion criteria. Ultimately, 51 full-text articles were assessed for eligibility, leading to the exclusion of 24 articles and the inclusion of 27 studies in the qualitative synthesis of this systematic review.

A total of 27 studies [46,47,48,49,50,51,52,53,54,55,56,57,58,59,60,61,62,63,64,65,66,67,68,69,70,71,72,73,74], 26 non-randomized control studies and 1 randomized control trial were analyzed, consisting of 7928 overweight/obese children and adolescents within the age range of 4.5–20 years who were at different stages of pubertal development. The interventions took place in ten countries (Brazil, Poland, China, USA, Germany, Denmark, Spain, Italy, Portugal, and Czechia), representing a variety of populations. Table 3 summarizes the general data extracted from each study. The interventions were multidisciplinary, having dietary, exercise, behavioral, and/or medical evaluation components, and their duration ranged from 4 weeks to 10 years. The specific elements of each lifestyle intervention are analytically presented in Supplementary Table S1. These studies examined the gene–environment interaction in obesity lifestyle interventions, considering the effect of SNPs in 92 different genetic loci on BMI or body composition improvement. Polymorphisms in 24 genes were significantly associated with BMI and/or body composition amelioration (Table 4, Supplementary Table S4), and the full set of genetic loci examined and the identified genetic associations are available in Supplementary Table S4. However, no studies were found through our literature search, testing the relation of CNVs with BMI/body composition alteration during childhood obesity management programs.

The assessment of validity showed that the included studies in the present systematic review are of high quality, since they were rated as good in the NOS scale (Supplementary Tables S2 and S3).

## 4. Discussion

In the present study, we systematically reviewed the literature to decode the gene–environment interactions with BMI and body composition alteration in obesity lifestyle interventions in children and adolescents, identifying significant associations with SNPs in 24 genetic loci. The main significant findings of the present study with respect to the effect of the genotype in the alteration of BMI and body composition after the implementation of pediatric lifestyle interventions are summarized in Table 4, while all of the examined genotypic associations are analytically presented in Supplementary Table S4.

### 4.1. Central Nervous System and Obesity

Among the assessed genes, the cell adhesion molecule 2 (*CADM2*; OMIM * 609938) gene encodes a synaptic cell adhesion molecule that is a member of the immunoglobulin superfamily, which is widely expressed in multiple areas of the central nervous system [77,78]. During a 4- to 6-week standardized in-hospital lifestyle intervention program in 1198 children and adolescents with excess adiposity, Heitkamp et al., showed that the G allele in rs13078960 SNP of *CADM2* was significantly associated with reduced BMI-SDS change [55]. The *CADM2* gene mediates synaptic organization and function and coordinates signaling networks in several brain areas, thereby contributing to the control of adiposity and energy balance [78,79,80]. Genome-wide association studies have identified SNPs in *CADM2* (rs13078960, rs1307880) as obesity susceptibility loci, similarly with its related family member *CADM1* [81,82]. Interestingly, the obesity risk allele in rs13078960 SNP leads to increased expression of CADM2 in multiple areas of the human brain (caudate basal ganglia, putamen basal ganglia, cerebellum, and hypothalamus), providing further support of the association of CADM2 with increased adiposity and its role in energy homeostasis [79,83]. Studies in rodent models have additionally shown that CADM2 is expressed in brown adipose tissue and skeletal muscles of high-fat-fed mice [79,83], while deficiency of CADM2 in mice is associated with a leaner phenotype. Moreover, research data highlight a leptin-mediated control of CADM2 expression, indicating an involvement of this protein in the leptin signaling pathway [79]. Exogenous leptin administration in obese and insulin-resistant mice counterbalanced the overexpression of CADM2 in the obese state in the brain, while loss of CADM2 resulted in increased leptin sensitivity [79]. The introduction of a low-carbon ketogenic diet in obese mice produced the same effects on CADM1 overexpression, with CADM1 being involved in the functions of CADM2 [83]. A treatment effect of CADM2, independent of leptin, on the signaling cascade was also observed, given that CADM2 knockout mice show increased levels of phosphoryliated-STAT3 in the hypothalamus [79]. Overall, the risk allele in rs13078960 SNP for *CADM2* predisposes to obesity [82] and incommodes the weight loss process in the pediatric population with overweight and obesity [55]. Further research into the role of *CADM2* during obesity lifestyle interventions is necessary, while the ketogenic diet approach seems to be a promising topic for research [83].

Furthermore, when assessing the dopamine receptor D2 (*DRD2*; OMIM * 126450) gene, Roth et al. showed that homozygous carriers of Τ allele in rs18000497 SNP near the *DRD2* gene exhibited decreased BMI-SDS reduction after a one-year outpatient obesity lifestyle intervention program [67]. The rs18000497 SNP or TaqIA, a restriction fragment length polymorphism, is located within exon 8 of the ankyrin repeat and kinase domain containing protein 1 gene (*ANKK1*; OMIM * 608774), possibly altering the substrate-binding specificity of ANKK1 protein [84]. This SNP is also in linkage disequilibrium with the *DRD2* gene, which is positioned more than 10 kb downstream and encodes the post synaptic dopamine DRD2 receptor [84]. In accordance to the findings by Roth et al., other research groups have identified an association of the minor T allele with a difficulty to improve BMI following interventions [85,86]. The rs18000497 plays a central role in the dopaminergic-associated mechanistic models [84]. To begin with, feeding behavior and energy expenditure are regulated by a complex interplay between overlapping homeostatic and reward neurocircuits, with the latter being mediated by multiple neurotransmitter systems, such as the dopaminergic, endocannabinoid, opioid, GABAergic, cholinergic, and serotonergic [87]. Dopamine (DA), a metabolite of tyrosine, acts as a hormone and a major neurotransmitter, mediating cognition, locomotor activity, emotional responses, endocrine functions, metabolic sensing, and the “gut-brain” axis interaction, thereby determining feeding behavior [32]. Dopamine deficiency in the dorsal striatum can result in starvation due to lack of motivational eating [88,89]. Overall, unhealthy dietary patterns and excess adiposity have been associated with disruption in dopaminergic signaling, impacting compulsive-like feeding, food preferences, nutrient sensing, glucose metabolism, and neurocognitive impairment, thus reflecting an association of alterations in the dopaminergic pathways with diet-induced obesity [84].

Other studies have revealed similar neuroadaptations in the DA pathway between food-seeking behavior and substance abuse, introducing a model of addiction to highly palatable food consumption, leading to obesity, in an underlying hypodopaminergic environment [32]. Interestingly, the presence of the minor T allele (TaqIA1) relates to diminished DRD2 receptor density in the brain, causing alterations in reinforcement and cognition learning [90]. Overall, an inverted U-shaped model of dopaminergic effect on cognition learning has been proposed, which varies based on the allele in the rs18000497 SNP [84]. In addition, a diet rich in fat was associated with blunted DA response in the striatum and reduced DRD2 levels [91], indicating a nutrient-specific control of the dopaminergic system [84]. TaqIA1 carriers, who have reduced DRD2 function, displayed significant weight gain following a highly palatable diet [92,93]. A relation of the downregulation of DR2D, such as in TaqIA1 carriers, with enhanced food intake is also evident given the role of DRD2 antagonists in the establishment of food-seeking behavior and the inhibition of the anorexic effects of leptin, further supporting the model of food addiction [32]. Moreover, a reduction in DRD2 levels is thought to reduce physical activity and the related energy expenditure, thus increasing adiposity, which further downregulates DR2D function, creating a futile cycle [94]. Regarding the underlying molecular mechanisms, chronic exposure to saturated lipids is recognized as a crucial mediator of DRD2, possibly through inflammatory pathways connecting astrocytes with DRD2 [84]. Chronic consumption of dietary fat generates inflammatory-like changes in the brain, partially mediated by NF-κΒ, resulting in transcriptional adaptations in DRD2 levels and signaling [84]. Carriers of the TaqIA1 may be more susceptible to these inflammatory responses [84]. Finally, TaqIA seems to interact with the *FTO* gene, one of the most strongly associated genetic loci with obesity, influencing dopaminergic pathways connected to obesity-related impaired learning functions, adiposity, and insulin resistance [84].

Another gene linking dopamine and serotoninergic models with the control of energy homeostasis is the LIM homeobox transcription factor 1-beta (*LMX1B*; OMIM * 602575), which encodes the LMX1B protein, a member of the LIM-homeodomain family of proteins [95]. In a multidisciplinary lifestyle intervention program conducted by Heitkamp et al. to address childhood obesity in more than one thousand children and adolescents, the homozygous state for the A allele in rs10733682 of *LMX1B* gene was shown to promote greater weight loss [55]. Conversely, Hollensted et al. associated the T allele in rs3829849 SNP of *LMX1B* with decreased BMI-SDS change after a family-centered multidisciplinary behavioral lifestyle intervention in 754 children and adolescents with excess adiposity [56]. These two studies examined different SNPs of the *LMX1B* gene and used different methodology designs [55,56]. Another study conducted in adults associated females homozygous for the AA genotype in *LMX1B* rs10733682 polymorphism with increased adiposity and revealed an interaction of this genotype with macronutrient intake and dietary patterns, resulting in increased triglycerides with fat and carbohydrate consumption and increased BMI and waist circumference with protein intake [96]. In addition, the obesity risk allele in rs10733682 SNP has been associated with reduced satiety responsiveness in the pediatric population [97]. The association of the rs10733682 and rs3829849 polymorphisms in *LMX1B* loci with obesity has been established by GWAS both in adult and pediatric populations [27,82] and may be possibly explained by the role of LMX1B in serotonergic and dopaminergic pathways [98,99]. LMX1B acts as a continuously expressed transcription factor at successive stages, regulating serotonin (5-HT) genesis and reuptake, as well as 5-HT neuronal axon primary formation, targeted routing, and terminal arborization [98]. Serotonergic signaling in the central and peripheral nervous system is a major mediator of food intake and energy homeostasis, taking part in the homeostatic and hedonic/reward pathways of feeding behavior [31]. Serotonergic pathways in the central nervous system are anorexigenic and promote energy expenditure by increasing thermogenesis in the brown adipose tissue [31], while 5-HT pathways in the peripheral nervous system promote energy storage by enhancing lipid anabolism [100]. Data from rodent models and humans reveal impaired serotonergic circuits in the obese state, with reduced 5-HT levels and attenuated signaling [31]. These changes occur early in overfeeding conditions with high-fat and -sugar diets [101,102]. Moreover, meal timing plays a crucial role in 5-HT regulation: during a 4-week hypocaloric diet, the striatal dopamine transporter and thalamic serotonin signaling increased when most caloric daily intake was consumed in breakfast and decreased when it was consumed in the evening [103].

The pleiotropic functions of leptin in energy homeostasis and metabolism are mediated through the leptin receptor, which is encoded by the leptin receptor gene (*LEPR*; OMIM * 601007) [104]. Two of the included studies examined SNPs in *LEPR* [49,52]. Firstly, polymorphisms of *LEPR* were studied by Gajewska et al. in prepubertal children undertaking a weight management program, in which carrying at least one minor G allele in Q223R together with the wild-type K665N led to the greatest weight loss, fat mass reduction, and increase in soluble leptin receptor levels [52]. Secondly, in a research study conducted by Corgosinho et al., adolescents carrying the C allele of rs2767485 in *LEPR* presented increased leptin concentrations and increased expression of orexigenic peptides NPY, AgRP, and MCH, a metabolic state constituting an obstacle to weight loss, with a concomitant increase of AgRP concentration after a one-year obesity lifestyle intervention [49]. On the other hand, TT homozygotes showed greater BMI change [49]. Adult female carriers of the T allele in *LEPR* Ser (T) 343 Ser (C) polymorphism showed a stronger predisposition to obesity, while carriers of the C allele demonstrated greater weight loss in response to a low-caloric diet intervention compared to non-carriers [105]. In addition, the Lys656Asn polymorphism in *LEPR* has been associated with variations in the response to lifestyle modifications in adults [106]. LEPR is a type I cytokine transmembrane receptor, signaling through a tyrosine kinase, the Janus kinase 2 (JAK2), and thus activating multiple molecular pathways [104]. Moreover, LEP–LEPR binding can lead to the control of energy homeostasis independently of tyrosine phosphorylation, which is possible through pathways involving phosphoinositide 3-kinase (PI3K) [104]. Leptin acts in the central nervous system through the long isoform of LEPR, being expressed in neuronal cells in multiple regions of the brain, and is responsible for feeding and energy balance [107]. The soluble leptin receptor, a short form of leptin receptor, binds the circulating leptin, preventing clearance [108]. Leptin is secreted by adipose tissue in relation to its stores of triglycerides and has pleiotropic actions in energy balance and adiposity regulation, in growth, thermogenesis, and glycemic control, the production of adrenal corticosteroids, and the function of the reproductive and immune system [104,108]. Leptin is a key component of the leptin–melanocortin pathway, exercising anorexic and lipolytic functions [104]. Interestingly, excess adiposity is characterized by hyperleptinemia [108], while weight loss is associated with decreased leptin concentrations [52].

Associated with the above-mentioned leptin pathway, the melanocortin 4 receptor gene (*MC4R*; OMIM * 155541) encodes a G-protein-coupled receptor with unique structure [109]. Homozygosity for the C allele in s17782313 of *MC4R* has been associated with greater BMI and body composition outcomes in an inpatient weight-reduction program by Zlatohlavek et al. in pediatric subjects with excess adiposity [73]. The C allele in s17782313 has also been associated with better BMI and body composition change as a part of genetic risk score (GRS) during a three-month multidisciplinary lifestyle intervention by Moleres et al. [61]. Finally, Vogel et al. showed that female carriers of at least one risk allele for the *MC4R* rs17782313 and rs12970134 polymorphisms demonstrated more efficient BMI-SDS reduction than males during a one-year multicomponent lifestyle intervention to manage obesity during childhood [71]. Exploring the nutritional interaction with rs17782313 SNP, regular consumption of meals (5 meals including breakfast versus ≤ 4 meals) in adolescence seem to attenuate the obesity risk from the risk alleles in *MC4R* rs17782313 in both genders and from *FTO* rs1421085 only in males, possibly because of more favorable postprandial thermogenic and glucose metabolism responses [110,111]. Moreover, an increased consumption of salt in *MC4R* rs17782313, *SEC16B* rs543874, and *KCTD15* rs11084753 risk allele carriers increases the risk of obesity by both increasing the intake of sugar-sweetened soft drink consumption and by extension the intake of calories and causing water retention and disruption of fat metabolism [112]. The MC4R is embedded in the leptin–melanocortin signaling pathway and is expressed in key brain areas [113], contributing significantly to energy homeostasis, appetite regulation, thermogenesis, and peripheral glucose metabolism [114]. The MC4R has important anorexigenic effects. The entire spectrum of MC4R genetic variations has been related to BMI heterogeneity [114]. More than 200 mutations have been identified so far in obese patients, ranging from rare loss of function mutations leading to extreme monogenic obesity to common variations connected with the pathogenesis of polygenic obesity [114]. The MC4R receptor is activated in the satiety state by the adrenocorticotropic hormone (ACTH) and the post-translational derivatives of POMC and the a-MSH, β-MSH, and γ-MSH neuropeptides; it is inhibited by orexigenic AgRP-mediated signaling, expressed in AgRP/NPY neurons in the arcuate nucleus [114]. The rs17782313 SNP is located 188 kb downstream of *MC4R* and has been strongly associated with the development of obesity in populations of different ethnicity and age, including children and adolescents [115,116,117,118]. Ligand-receptor changes could possibly contribute to the positive effect of the minor allele of rs17782313 SNP in BMI reduction in childhood obesity lifestyle interventions [71]. On the other hand, gain-of-function mutations in MC4R protect from obesity and its cardiometabolic comorbidities, possibly through a signaling bias toward β-arrestin recruitment [119], causing an increased MC4R expression in the cell surfaces [119]. Overall, the obesity risk allele in the *MC4R* rs17782313 may beneficially affect the decrease of adiposity in short-term pediatric therapeutic interventions, either individually or as part of a GRS, possibly through a sex-specific interaction.

The melanocortin 3 receptor gene (*MC3R*; OMIM * 155540) encodes the MC3R, a G-protein-coupled receptor, which is a part of the melanocortin system and a key component of energy homeostasis, adiposity, and food-intake regulation, participating in appetite control and eating-induced motivational responses in situations of nutrient restriction and negative energy equilibrium, as well as glucose metabolism [120,121]. Santoro et al. examined the role of C17A (Thr6Lys) and G241A (Val81Ile) polymorphisms of *MC3R* gene in a multicomponent weight reduction intervention, associating carriers 6Lys and 81Ile alleles with reduced BMI change [68], in accordance with studies in adults [122]. The MC3R signaling leads to inhibition of POMC in the central nervous system, while it is also expressed in peripheral organs, such as heart, kidneys, liver, gut, and adipose tissue [68]. In mice, *MC3R* deficiency has been associated with the obese phenotype with Cushing-like characteristics, such as increased cortisol levels and visceral fat, and to an unbalanced metabolic rate and profile with reduced lipolysis [121]. In *MC3R* dysfunction, obesity presents in milder levels compared with *MC4R* deletion, while a coexistence of these two deficiencies has a synergic effect on increased adiposity [121]. In addition, increased anthropometric measures of adiposity, such as BMI and fat mass, have been observed in the homozygous state for T6K + V81I in pediatric population studies [123,124,125]. The T6K +V81I haplotype is by far the most well-studied; its phenotypic effects are most prominent in the homozygous state, with milder clinical effects seen in heterozygous carriers [121]. The haplotype’s effects may be more pronounced in infancy and childhood [121]. In addition, both human and rodent models suggest that T6K + V81I carriers have lower rates of lipid oxidation, combined with increased oxidation of glucose [121], possibly explaining the difficulty to lose weight during interventions [68].

### 4.2. Adipose Tissue and Obesity

Among the genes associated with adipose tissue formation, metabolism, and obesity during childhood, Heitkamp et al. showed that children/adolescents homozygous for the G allele in the rs12940622 polymorphism of the regulatory-associated protein of *MTOR* (*RPTOR*; OMIM * 607130) gene are resistant to weight loss compared to homozygous non-carriers in a 4–6-week obesity intervention program [55]. The *RPTOR* gene encodes an evolutionarily conserved protein, which forms a stoichiometric complex with the mechanistic target of rapamycin (mTOR) [126]. The mTOR signaling pathway modulates fundamental cellular processes, and its dysregulation contributes to the pathogenesis of tumors, metabolic diseases, neurodegeneration, and aging [126]. The relation of the rs12940622 SNP of the *RPTOR* gene with excess adiposity has been identified in large GWAS [82] and is possibly explained by the crucial role of RPTOR in the functions of the mTORC1 pathway [55]. The mTORC1 pathway integrates nutritional and hormonal signals, orchestrating anabolic and catabolic processes and enabling the transition between them [126]. Indeed, growth factors, insulin, amino acids adequacy, such as in feeding conditions, oxygen, and ATP availability promote mTORC1 anabolic signaling [126]. Protein synthesis, lipogenesis, and nucleotide biosynthesis are enhanced, and energy homeostasis is regulated [126]. Simultaneously, mTORC1 suppresses catabolism and autophagy [126]. 

In contrast, in a nutrient-deprived environment, under energetic stress, hypoxia, DNA damage, or rapamycin therapy, mTORC1 is inhibited, thus promoting catabolic pathways towards autophagy and lysosome biogenesis [126]. In this way, cellular amino acids can be replenished, reactivating mTORC1 and establishing an important feedback mechanism for survival between the lysosomes and mTORC1 [126]. Interestingly, hepatic RPTOR deficiency in mice attenuates lipogenesis in the liver [126]. In rodent models, RPTOR deletion in adipocytes diminishes white adipose tissue (WAT) and promotes lipolysis and gradual lipodystophy with a parallel lipid accumulation in the liver, developing hepatic steatosis, hepatomegaly, and insulin resistance [127]. Interestingly, these RTOR knockout mice have a lean phenotype and present hyperphagia with a resistance to diet-induced obesity [127,128]. Nevertheless, RPTOR depletion, inhibiting the mTORC1 pathway, has been associated with an expansion of lifespan in multiple organisms [126]. Overall, the association of RPTOR/mTORC1 with adiposity regulation becomes evident [126]. In overfeeding conditions with a surplus of nutrient and hormonal signals, the mTOR pathway is overactivated, contributing to the pathogenesis and maintenance of many diseases, such as obesity, diabetes mellitus type 2, and metabolic syndrome [126]. Research towards developing mTORC1 specific inhibitors could be promising in the treatment of obesity [126]. Some researchers have suggested a U-shaped inverted model in the association of mTORC1 function with adiposity, where optimal mTORC1 signaling is necessary for pro-adipogenic and lipogenic effects [129].

The FTO alpha-ketoglutarate-dependent dioxygenase gene (*FTO*; OMIM * 610966) encodes the namesake nuclear protein, which belongs to the AlkB-related non-heme iron and 2-oxoglutarate-dependent oxygenase protein superfamily and is ubiquitously expressed in the body, with the highest concentrations found in the brain and the pancreatic islets [130,131]. Barbian et al. [48], do Nascimento et al. [51], Moraes et al. [63], and Müller et al. [64] studied the rs9939609 SNP of *FTO*, Hollensted et al. [56] studied the rs1421085 SNP of *FTO*, Scherag et al. [69] studied the rs1558902 SNP of *FTO*, and Schum et al. [70] studied the rs1421085, rs17817449, and rs9939609 SNPs of *FTO* and showed that these variants did not modify BMI or body composition following lifestyle interventions for management of childhood obesity. Conversely, Moleres et al. [61] found a greater BMI reduction after a 3-month multidisciplinary lifestyle intervention in A-allele carriers of rs9939609 SNP both individually and as a part of a GRS. Moreover, Reinehr et al. [66] described a synergistic effect in the homozygous state of the C allele in rs7566605 SNP of *INSIG2* together with the A allele in rs9939609 SNP of *FTO*, leading to reduced BMI change after a one-year outpatient lifestyle intervention. In addition, Zlatohlavek et al. [73] associated the homozygosity for the G allele in rs17817449 SNP of *FTO* genetic loci with greater BMI change both individually and in synergy with the CC homozygosity in rs17782313 of *MC4R* during a 4-week inpatient weight reduction program. Finally, Hagman et al. [54] associated the A-allele homozygosity in rs8050136 SNP of *FTO* with greater BMI reduction after a 12-month to 10-year clinic-based behavioral modification therapy aimed at children and parents. The *FTO* gene mediates oxidative demethylation of various RNA forms and takes part in fat mass regulation, adipocyte differentiation, and energy homeostasis [131]. The genetic loci of *FTO* emits one of the strongest signals reported in GWAS related to obesity and energy balance, implicating FTO in the pathogenesis of excess adiposity, while mutations of the gene cause growth retardation, developmental delay, and facial dysmorphism [130]. Claussnitzer et al. in 2015 elucidated the underlying pathophysiological mechanisms associating *FTO* with obesity development by identifying its effect on the IRX3 gene promoter, located 0.5 MB downstream of *FTO* [132]. The T-to-C allele substitution in the rs1421085 SNP of *FTO* impairs the binding of the ARID5B transcriptional suppressor, lifting its inhibitory effect on a major preadipocyte enhancer [132]. In this way, the expression of IRX3 and IRX5 is doubled in the early stages of adipocyte differentiation, and a cell-cycle-independent turnover of beige adipocytes to white takes place, leading to a reduction of mitochondrial thermogenesis, a process regulated by UCP1, PGC1α, and PRDM16, and to an increase in lipid storage [132]. Therefore, a conversion of the biological processes from energy expenditure to energy storage is reflected [132].

Another gene associated with adipogenesis and possibly the low-grade inflammation of obesity is the ETS protooncogene 2 transcriptional factor gene (*ETS2*; OMIM * 164740), which encodes the ETS2 proto-oncogene and transcription factor, which specifically recognizes and binds to the core motif DNA sequence GGAA/T of target genes [133]. In their study, Heitkamp et al. showed a significantly greater reduction in body weight and BMI in children/adolescents homozygous for the effect C allele in rs2836754 SNP of the *ETS2* gene compared to non-carriers during a 4–6-week in-hospital lifestyle intervention [55]. The ETS2 plays a fundamental role in a broad spectrum of cellular processes, regulating development, proliferation, differentiation, migration, transformation, and apoptosis [133]. The rs2836754 SNP association with susceptibility to the obese phenotype has been recognized in GWAS [82]. ETS2 is a transcriptional regulator of early adipocyte differentiation both in vivo and in vitro [134]. Furthermore, ETS2 has a diverse role in inflammatory processes [135], potential affecting the inflammatory state of obesity. ETS2 has been identified as a transcriptional regulator of many cytokines, such as IL-5 and the p-40 subunit of IL-12 [136]. Animal models show that in acute inflammation, ETS2 has anti-inflammatory effects, acting as a negative regulator of lipopolysaccharides and VSV-induced inflammation by directly binding to the IL-6 promoter, inhibiting transcription and inhibiting MAPK/NF-κB signaling [135]. Moreover, ETS2 regulates miR-155 inflammatory production both positively and negatively by an IL-10 mediated inhibition [137]. On chronic inflammation, ETS2 is essential to persistent TNF-a production [138].

Another gene associated with adipogenesis is the lysine acetyltransferase 8 (*ΚAΤ8*; OMIM * 609912) gene. The A allele in rs9925964 SNP of the *ΚAΤ8* gene was associated with greater BMI-SDS reduction in a 4–6-week in-hospital lifestyle intervention for the management of obesity in childhood conducted by Heitkamp et al. [55]. The *KAT8* gene encodes the catalytic subunit of two multiprotein complexes (male-specific lethal-MLS and non-specific lethal-NSL) [139]. The produced protein is a member of the MYST histone acetylase family, also known as MYST1, which was first identified as males-absent-on-the-first (MOF) in Drosophila melanogaster [139]. Overall, KAT8 plays a crucial role in multiple physiological functions, such as gene transcription control, chromatin architecture maintenance, cell cycle regulation, autophagy, early embryogenesis, mitochondrial transcription, and DNA damage repairment, while its dysfunction has been associated with tumorigenesis and defects in these fundamental processes [139,140,141,142]. Findings from GWAS have highlighted rs9925964 SNP in *KAT8* gene as a risk locus for obesity [82], an association possibly explained by data associating KAT8 with adipose tissue development and function [143,144,145], central control of metabolism, and diet-induced obesity [146]. Furthermore, studies in *KAT8* haploinsufficient mice have shown that KAT8 is an important epigenetic regulator of carbohydrate, amino acid, and adipose tissue homeostasis [146]. In addition, other pathways except glucose metabolism are potentially affected, such as brain and heart vitamin B6 metabolism [146]. Another study by Brenachot and colleagues introduced KAT8 as a mediator of energy balance in the brain through the regulation of polysialylation, a necessary component of synaptic plasticity in feeding pathways [147]. Using mice with *KAT8* total depletion in the mediobasal hypothalamus, *St8sia4* gene transcription was inhibited and polysialic acid levels were reduced, leading to increased feeding behaviors and an obese phenotype [147]. Small molecule inhibitors of acetylation in a cell-specific manner could constitute a future research field in obesity treatment [146].

The transmembrane protein 18 gene (*TMEM1*; OMIM * 613220) is an evolutionarily highly conserved gene, encoding the nuclear transmembrane protein 18 [148], which mediates the migration process of neural precursors to glioblastoma cells [149]. In a 3-month multidisciplinary lifestyle intervention by Moleres et al., the risk G allele in rs7561317 SNP of *TMEM18* was associated with greater BMI-SDS and fat mass reduction both on its own and as a part of a high GRS score [61]. Conversely, the rs4854349 SNP studied by Hollensted et al. in a 6–24-month family-centered multidisciplinary behavioral lifestyle intervention [56], the rs4854344 SNP studied by Zlatohlavek et al. in a 4-week in-patient weight reduction program [74], and the rs11127485 SNP studied by Scherag et al. in a one-year out-patient lifestyle intervention program [69] did not have significant associations with BMI or body composition change. The *TMEM18* is a preserved gene and has a well-established role among genes involved in obesity pathogenesis [150]. Various polymorphisms in the *TMEM18* loci have been implicated in obesity through GWAS and observational studies, an association more pronounced in the pediatric population [81,82,150,151,152,153,154,155,156]. Transmembrane protein 18 is ubiquitously expressed in most cells in the body, including the adipose tissue, the hypothalamus, and the brainstem, which are major modulators of energy homeostasis [150,155,157]. Interestingly, Rask-Andersen et al. associated body weight with the expression of TMEM18 in mice prefrontal cortex, which regulates executive functions and behavior characteristics [156]. Trying further to understand the pathophysiological mechanisms of TMEM18-mediated obesity, Landgraf and colleagues in 2020 turned their research towards adipose tissue metabolism instead of the central nervous system [155]. They showed that the expression of TMEM18 varies in adipose tissue of children without excess adiposity, depending on the presence of the obesity risk allele in rs7561317 and rs17729501 polymorphisms [155]. Moreover, TMEM18 expression in adipocytes is attenuated in children [155] and adults [158] with obesity, as well as with obesity-induced adipose tissue and metabolic dysregulation, such as in insulin resistance. However, an association with eating behavior traits was nοt confirmed [155], in accordance with data from a cohort in Greek pediatric population [156]. In addition, TMEM18 was identified as an enhancer of *PPARG1* promoter-induced transcription, exercising a major role in PPARG1-dependent adipogenesis [155]. In the inflammatory state of obesity, *TMEM18* downregulation reduces PPARG1 levels, contributing to the metabolic dysregulation, adipocyte hypertrophy, adiponectin reduction, impaired glucose, and lipid metabolism and obesity aggravation [155].

Another significant finding from the study by Moleres et al. (2012) was the evidence that children with a higher obesity risk GRS score, constituting the obesity risk G allele of rs1801282 SNP in the peroxisome proliferator activated receptor gamma gene (*PPARG*/*PPARγ*; OMIM * 601487) alongside SNPs in *FTO*, *MC4R*, *TMEM18*, *IL6*, and *ADIPQ* had greater BMI and fat mass at the beginning together with a greater BMI and fat mass reduction after a 3-month lifestyle intervention, whereas children with lower GRS presented a greater improvement in their metabolic profile after intervention [61]. The *PPARγ* gene encodes a member of the nuclear receptor superfamily PPARs, the PPARγ or PPARG, and is mainly expressed in adipose tissue (white and brown), as well as in other organs such as macrophages, endothelium, liver, colon, muscles, and kidneys [159]. It has multiple different isoforms, created through alternative transcription and splicing, including PPARγ1 and PPARγ2, with the latter being mainly expressed in the adipose tissue [160,161]. PPAR gamma presents pleiotropic functions, taking part in a variety of biological processes, including adipogenesis, glucose metabolism, lipid oxidation and storage, control of autophagy, and inflammatory responses, and has therefore been implicated in the pathological state of obesity, type 2 diabetes mellitus, atherosclerosis, cancer, and Familial Partial Lipodystrophy Type 3 [159,162]. Among the natural agonists of PPARγ are the polyunsaturated fatty acids, glucocorticoids, and insulin [35], with thiazolidinedione also having a high affinity for PPARγ, which exercise their therapeutic effects in type 2 diabetes mellitus [159,163]. Data show that caloric restriction antagonizes PPARγ, whereas dietary fat activates it [164]. Genetic variations in PPARγ have been associated with BMI distribution from birth to adulthood in Caucasians, possibly in a sex-specific manner, including the rs1801282 SNP, also known as Pro12Ala [161,164,165]. PPARγ is characterized as the master regulator of adipogenesis [35]. PPARγ deficiency in rodents leads to hepatic steatosis and lipodystrophy, since there is no other component to replace the role of PPARγ in adipogenesis [166]. A high PPARγ activity induces the process of hyperplasia during adipogenesis, while under a low PPARγ drive, the adipogenesis dynamic is impaired, with the existing adipocytes becoming hypertrophic [35]. This expansion in size impairs the function of PPARγ and limits the adipogenetic turnover of mesenchymal cells to adipocytes, while it favors mechanical-stress-induced hypoxia, the low-grade inflammation of obesity, and insulin resistance [35]. Acute inflammation may promote tissue remodeling through the enhancement of adipogenesis [35]. However, in chronic inflammation like that in obesity, PPARγ expression can be downregulated, and the protein is degraded [167]. The rs1801282 SNP, located in exon B, creates an amino acid change of proline to alanine (Pro12Ala) in the N-terminus of the PPARγ2 isoform [161]. This substitution has been associated with reduced binding affinity to the receptor of target genes [161], attenuating the transcriptional functions of PPARγ, and with impaired recruitment of tissue-specific cofactors, which dysregulate its role in adipogenesis and the different expression patterns in different cell types and increase insulin sensitivity [168]. Indeed, carriers of the Pro allele have an increased risk of developing diabetes mellitus type 2 [169]. Considering the functions of PPARγ in metabolism, this amino acid change may explain the findings in Moleres et al. study [61], but further research is needed on its effect on obesity lifestyle interventions. Interestingly, a gene–environment interaction has been described between rs1801282 SNP and nutrition, where BMI and insulin concentration are inversely associated with the polyunsaturated to saturated fatty acids ratio of the diet in carriers of the obesity G risk allele [170].

The sonic hedgehog (SHH) signaling and ciliogenesis regulator SDCCAG8 (*SDCCAG8*; OMIM * 613524) gene encodes a protein associated with the centrosome, which may regulate centrosome during interphase and mitosis [171]. Scherag et al. studied variations in five (*FTO*, *MC4R*, *TMEM18*, *SDCCAG8*, *TNKS*/*MSRA*) genetic loci and identified an association of the homozygosity for three intronic SNPs (rs10926984, rs12145833, rs2783963) in *SDCCAG8* with reduced BMI change in children/adolescents undergoing a one-year multidisciplinary obesity lifestyle intervention, although similar correlations were nοt identified in an adult sample undergoing an hypocaloric diet intervention [69]. The SDCCAG8 protein is essential for ciliogenesis through its modulatory role on RABEP2-dependent centrosomal localization, as well as for the optimum activation of multiple signaling pathways, such as the Hedgehog pathway, which require fully functional cilia [172]. It also contributes to the creation of cell polarity and the epithelial lumen [172]. Mutations of SDCCAG8 are implicated in the pathogenesis of retinal–renal ciliopathies [171]. Bardet–Biedl Syndrome, one of the major causes of syndromic obesity, and Senior–Loken Syndrome 7 are associated with mutations in SDCCAG8 [171]. Moreover, polymorphisms of *SDCCAG8* gene, such as the ones mentioned above, have been implicated in the development of obesity through GWAS [173]. Indeed, both deleterious mutations and common polymorphisms in genes regulating ciliary related proteins have been associated with all obesity phenotypes, from monogenic and syndromic to multifactorial polygenic obesity [174].

Children/adolescents carrying the A allele in rs11170468 SNP of copine 8 (*CPNE8*) gene were resistant to BMI-SDS reduction in a 4–6 week obesity lifestyle intervention program conducted by Heitkamp et al. [55]. The *CPNE8* gene encodes the copine 8 calcium-dependent membrane-binding protein, which takes part in calcium-mediated molecular events and interactions in cell membranes and the cytoplasm [175]. Calcium is an important molecule in various cellular processes [175]. Obesity has been associated with dysregulation of cytoplasm and organelle Ca^2+^ homeostasis and the related signaling transmission [176]. Excess adiposity and overfeeding leads to Ca^2+-^mediated disruption of hepatic glucose metabolism, mitochondrial dysfunction, endoplasmic reticulum stress, and autophagy in liver and immune-cell dysfunction [176]. Moreover, in adipose tissue, Ca^2+^ contributes to the regulation of adipogenesis, lipid synthesis, and thermogenesis in brown adipocytes [176].

### 4.3. Adipose Tissue Metabolism

Moleres et al. in 2014 studied genes implicated in lipid and energy metabolism and associated the minor allele in rs670 (−75 G/A) apolipoprotein A1 gene (*APOA1*; OMIM * 107680) with increased BMI and other measures of body composition, as well as with greater weight and BMI reduction after a 10-week intervention, while a combined analyses with the rs1800777 SNP in *CETP* explained up to 24% of BMI-SDS amelioration [62]. The *APOA1* gene encodes apolipoprotein A-I, the most abundant component of high-density lipoprotein cholesterol (HDL), and has antiatherogenic and anti-inflammatory actions [177,178]. Apolipoprotein A-I also promotes energy expenditure and modulates body fat content, lipolysis, and glucose metabolism, exercising an anti-obesity effect [177,178]. Indeed, *APOA1* knockout mice significantly increase their body weight and body fat accumulation despite a restriction in caloric intake because of an attenuation of lipolytic activity [179]. Conversely, *APOA1* transgenic mice or mice treated with the ApoA-I mimetic peptide D-4F, when fed a highly palatable diet, showed a reduction in adipose tissue, increased insulin sensitivity, and enhanced energy expenditure [177,178]. In addition, APOA1 promotes the β-adrenergic stimulated lipolysis and is speculated to participate in the differentiation of pre-adipocytes [177]. The common polymorphism rs670 is located 75 bp upstream from the transcriptional site of *APOA1* gene and may contribute to the variable gene environment interactions affecting metabolism [180]. Indeed, the A allele has been associated with an improvement of anthropometric measures, lipid profile, and insulin sensitivity following a hypocaloric diet in patients with obesity [181,182]. Homozygotes for the G allele have a higher risk of metabolic syndrome, possibly explained by their increased adiposity and insulin resistance [183].

Another genetic variant associated with lipid metabolism assessed in the study by Moleres et al. is the minor allele in rs1800777 (R451Q) cholesteryl ester transfer protein gene (*CETP*; OMIM * 118470), which was correlated with increased BMI and other measures of body composition and greater weight and BMI reduction after a 10-week multidisciplinary lifestyle intervention, while a combined analyses with the rs670 SNP in *APOA1* explained up to 24% of BMI-SDS reduction [62]. The *CETP* encodes a glycoprotein responsible for the exchange of lipids, such as cholesteryl ester and triglycerides, among lipoprotein particles, as well as the cholesterol efflux [184]. CETP regulates the transfer of esters from HDL to Apolipoprotein B (ApoB)–containing lipoproteins (VLDL, LDL) in exchange for triglycerides, thus affecting HDL particle size [184]. CETP deficiency leads to increased HDL concentrations and has an antiatherogenic effect [185]. Therefore, CETP inhibitors may be promising for the prevention of cardiovascular disease, since they increase HDL and decrease LDL and ApoB [185]. Polymorphisms in *CETP* affect the lipid profile and influence the risk of developing coronary heart disease [185,186]. Moreover, variations in this gene are associated with an alteration of the lipid profile in response to dietary fat intake, revealing a gene–nutrient interaction [187]. The SNP in the *CETP* (rs1800777) gene may affect lipid metabolism [188]. The presence of the minor allele has been associated with increased risk of developing central obesity, increased fat mass, waist circumference, and waist-to-hip ratio [189], as well as reduced HDL concentrations [189,190,191,192], enhanced CETP activity [191,193], and increased intimal wall thickness of the carotid arteries [191,194].

The lipoprotein lipase gene (*LPL*; OMIM * 609708) [195] encodes a lipoprotein lipase, a member of the lipase gene family, which catalyzes the hydrolysis of triglycerides in chylomicrons and very low-density lipoproteins (VLDLs), contributing to lipoprotein uptake in the tissues, the exchange of lipids between lipoproteins, and the lipoprotein-independent uptake of lipoprotein-derived lipids and lipophilic vitamins [196]. In the study by Gao et al., the rs283 SNP in *LPL* gene was associated in the homozygous GG allele carriers with greater body fat reduction and triglyceride concentrations and improvement in HOMA-IR following a 4-week aerobic training [53]. The authors attributed their findings to metabolic changes occurring during exercise, in which fatty acids are translocated from adipose tissue to muscles, where the rate of fat oxidation is increased [53]. A catecholamine-mediated increase of cAMP and an increase in intracellular calcium through muscle contraction were observed during exercise in skeletal muscles, upregulating *LPL* gene expression and increasing the hydrolyses of triglycerides to fatty acids [53]. Conversely, the exercise-mediated reduction of insulin downregulated LPL expression and activity in adipose tissue, attenuating the triglyceride absorption in adipose tissue [53]. The rs283 polymorphism is located in intron 6, and the authors speculated that the G allele can enhance *LPL* gene expression, increase LPL activity, and positively affect exercise-induced metabolic and adiposity changes [53].

In the studies performed by Reinehr et al. in 2008 [65] and 2009 [66], a statistically significant lower BMI and BMI-SDS reduction was noted in homozygous CC carriers in rs7566605 SNP of insulin-induced gene 2 (*INSIG2*; OMIM * 608660) gene during obesity lifestyle management interventions, while the 2009 study highlighted a synergistic effect in reduced BMI change after intervention in combined homozygous carriers of the CC genotype in rs7566605 SNP of *INSIG2* and the AA genotype in rs9939609 SNP of *FTO*. The *INSIG2* gene encodes the INSIG2 oxysterol-binding protein, which binds to the SREBP cleavage-activating protein (SCAP), retaining the SREBP/SCAP to the endoplasmic reticulum and inhibiting its transportation to the Golgi, while it also promotes the ubiquitination and degradation of HMGCR, negatively regulating lipid biosynthesis [197,198]. The association of the rs7566605 SNP of *INSIG2* [199] with predisposition to obesity is controversial [200], with data supporting this correlation in adults with severe obesity [201]. In the pediatric population, some studies have identified an association of the rs7566605 SNP with predisposition to obesity [153], while others have found such an association only when diet combined with physical exercise are taken into account [202]. Interestingly, in a pediatric cohort, homozygosity for the obesity risk C allele was associated with higher glucose concentrations, possibly introducing an indirect role of INSIG2 in promoting adiposity, through its effect on glucose metabolism [203]. Indeed, *INSIG2* gene has been implicated in diabetes pathogenesis [204,205].

Deram and colleagues identified two different patterns that link pediatric obesity, insulin resistance, and lipid metabolism for the perilipin 1 gene (*PLIN1*; OMIM * 170290) variations in children and adolescents [50]. Firstly, the T allele in rs1052700 was associated with improved BMI and body composition outcomes and lower HOMA-IR levels [50]. Secondly, the minor A allele in rs894160 was related to a high-risk metabolic profile in the pediatric population, observing an impaired glucose metabolism both at baseline and after intervention, and a higher prevalence of metabolic syndrome, without correlating this genotype with BMI and its alteration [50]. *PLIN1* is located on chromosome 15q26, a chromosomal location connected with obesity, impaired glucose metabolism, and hypertriglyceridemia [206,207]. Genetic variations in *PLIN1* gene have been associated with human disease [206,207]. The encoded perilipins are a family of phosphorylated proteins, highly conserved through species, encircling intracellular lipid storage droplets in adipocytes, steroid producing cells, liver, heart, and muscle cells [206,207,208]. They play a primary role in the regulation of lipid, glucose, and energy homeostasis through the formation and mobilization of adipocyte stores [206,207,208]. The *PLIN1* (rs1052700) is an obesity risk factor [209,210,211], which is also associated with better weight loss outcomes [50,212]. Indeed, the minor alleles in rs2304795 and rs1052700 are associated with body fat, waist circumference, and obesity risk in Caucasian adult women [209]. A gender-specific interaction and an ethnicity-dependent intragenic linkage disequilibrium (LD) structure (in Asians, rs1052700 and rs894160 are in LD, while rs2289487 and rs894160 are in LD in Whites) in the PLIN1 locus may affect the different associations in different populations [213]. 

In addition, the T allele in rs2304795 predisposes to the development of obesity in adolescence [211]. Examining the effect of rs1052700 polymorphism in response to diet modification, Jang et al. showed that carriers of the minor allele in rs894160 or rs1052700 of Korean origin showed a more significant decrease in waist circumference, fat mass, and free fatty acids, indicating increased lipolysis [206], during an energy-restricted weight loss intervention [212], similar to findings in Deram et al. [50]. Moreover, the rs894160 and rs1052700 were associated with a gene–diet interaction between increased saturated fatty acids and carbohydrate dietary intake and insulin resistance in Asian adult women [214]. In contrast, homozygotes for the minor T allele in rs1052700 had lower body weight and fat mass both at baseline and after a 6-week dietary weight loss intervention program [215], as shown by Deram et al. [50]. Interestingly, the minor A allele of rs894160 has been associated with prevention of obesity [215,216,217] but with resistance to weight loss following intervention [216,218]. In Deram et al., children were introduced to a balanced but not restrictive dietary education program, consisting of 1800 kcal/day [50]. The authors concluded that despite the association of the minor A allele in rs894160 with lower BMI in the literature [215,216,217], this protective effect is removed in the obese state, resulting in a metabolic unbalance and increased circulating non-esterified fatty acids, predisposing to a worse metabolic profile, insulin resistance, and type 2 diabetes [50]. Other research findings support the association of perilipin variations with diabetes risk, modified by central obesity in females [219]. Moreover, in the adipocytes of women with obesity homozygous for the A allele in rs894160, increased basal and noradrenaline-induced lipolysis combined with significantly lower levels of perilipin content have been identified, constituting a risk for type 2 diabetes [220].

The adipocyte-, C1q-, and collagen-domain-containing gene (*ADIPOQ*; OMIM * 605441) encodes the major adipokine, adiponectin [221]. The rs266729, rs16861194, rs822395, rs2241766, and rs1501299 SNPs were not associated with BMI change when examined individually in a 3-month outpatient multidisciplinary intervention program by Gajewska et al., nor in a 3-month multidisciplinary lifestyle intervention by Moleres et al. [52,61]. However, Moleres et al. associated a higher GRS, including the obesity risk alleles in the rs822395, rs2241766, and rs1501299 polymorphisms of *ADIPOQ*, with greater BMI and fat mass reduction [61]. Decreased concentrations of adiponectin were observed in overweight and obesity, dysregulating glucose, and lipid metabolism, contributing to the pathogenesis of type 2 diabetes and cardiovascular disease [222]. Interestingly, the rs266729 polymorphism of the adiponectin gene promoter variant has been correlated with decreased concentrations of adiponectin, predisposing to obesity and coronary atherosclerosis [223]. Considering the crucial roles of adiponectin in energy homeostasis and the pathophysiology of obesity, even in childhood and early puberty [104,224], interventions of longer duration and in larger population samples of children and adolescents are necessary to further investigate its associations with BMI improvement.

### 4.4. Adipose Tissue Inflammation and Obesity

Recent studies have highlighted the importance of low-grade aseptic inflammation in the pathogenesis of obesity [39]. In the study by Heitkamp and colleagues, homozygous carriers of the G allele of the rs13201877 polymorphism of the interferon-gamma receptor 1 (*IFNGR1*; OMIM * 107470) gene, when compared to homozygous non-carriers, showed significantly greater reduction in weight and BMI during a 4–6-week pediatric obesity intervention program [55]. The *IFNGR1* gene, located in chromosome 6q23.3, encodes the ligand-binding chain (alpha) of the gamma interferon receptor [225,226]. The gamma interferon receptor constitutes a heterodimer of IFNGR1 and IFNGR2 that is expressed in almost every cell type and exerts important antimicrobial, antiviral, and antitumor functions through its role in immune cell activation and the enhancement of antigen presentation [225,226]. The association of the rs13201877 polymorphism of *IFNGR1* gene with the risk of obesity development has been well-established in large GWAS [82]. Interferons are major cytokines, regulating anti-viral and autoimmune responses through cellular adaptations in energy homeostasis, protein and lipid metabolism, and cell cycle, structure, and metabolism [40]. In the acute phase of viral infections, interferons have an antiviral and immune stimulatory function, possibly by suppressing protein and lipid metabolism and reducing energy flow and lipogenesis [40]. On the other hand, chronic viral infection induces a prolonged but attenuated IFN activation, promoting immune dysfunction, obesity-related immune suppression, low-grade inflammation, and adipogenesis [40]. Furthermore, under chronic stimulation, IFNs can reprogram cellular lipid synthesis and transport, thus enhancing adipogenesis [40]. IFN-γ also contributes to the metabolism of ceramides (sphingolipid family), a biomarker of insulin resistance and adiposity [40]. The association of IFN-γ signaling with excess adiposity is further supported by the fact that treatment with IFN-γ results in decreased insulin sensitivity and inhibition of differentiation of pre-adipocytes to mature cells, thereby exerting an antiadipogenic effect, possibly through JAK-STAT1-mediated cascades [38,227].

Heitkamp et al., in their 4–6-week multidisciplinary lifestyle intervention, also demonstrated a lesser BMI-SDS reduction in carriers of the risk allele in rs13107325 SNP of the solute carrier family 39, member 8 (*SLC39A8*; OMIM * 608732) gene [55]. The *SLC39A8* gene is a highly evolutionarily conserved gene in vertebrates and a member of the solute-carrier gene superfamily, encoding the transmembrane transporter of cation ZIP8 [228]. ZIP8 is ubiquitously distributed throughout the body, mainly expressed in cell-surface membranes, but also in lysosomal, endoplasmic reticulum, and mitochondrial membranes [229]. ZIP8 plays a crucial role in cell processes, such as cell morphology, cytoskeleton formation, adhesion, migration, and proliferation, and exerts multiple pleiotropic functions both during embryogenesis and later in life. [229]. GWAS have associated *SLC39A8* gene variations with pathologies in multiple systems, such as dysmorphogenesis and immune, cardiovascular, gastrointestinal, coagulation, musculoskeletal, central nervous system, eye, kidney, and lung disorders [229]. ZIP8 is regulated by lipopolysaccharides, cytokines (such as TNF-α, IL-1β and IL-5), glucose, estrogen, and the concentrations of Zn^2+^ and Fe^2+^ [230]. The rs13107325 polymorphism is a nonsynonymous SNP located in exon 8 of *SLC39A8* gene and results in an amino acid change of hydrophobic alanine (major allele) to hydrophilic and polar threonine (minor allele) [230,231]. The carrier state of T allele has been associated with more than 20 pathological traits, including increased BMI, reduced HDL concentrations, coronary artery disease, hypotension, smoking-induced atherosclerotic plaques, higher risk of cardiovascular death, liver inflammation and fibrosis, inflammatory bowel disease, allergy, schizophrenia, low stature, and adolescent idiopathic scoliosis [229]. ZIP8 exerts a zinc-mediated protection from the toxic effects of TNF-α in lung epithelial cells early during inflammation [229]. Obesity is characterized by decreased plasma zinc concentrations [232], while weight loss results in an increase in plasma zinc concentrations [233]. The role of zinc in body weight regulation is further supported by the results of a recent meta-analysis in which zinc supplementation was associated with a decrease in body weight in individuals with overweight/obesity but who were otherwise healthy [234]. Overall, disruption of zinc homeostasis has been associated with oxidative stress, inflammatory processes, dyslipidemia, and diabetes mellitus type 2, mostly through mechanisms mediated by the rs13266634 polymorphism in the *SLC30A8* gene encoding zinc transporter ZnT8 [232].

The interleukin 6 gene (*IL6*; OMIM * 147620) encodes the major pro- and anti- inflammatory cytokine IL6, which binds to interleukin 6 receptor alpha (IL6R) and signals through the receptor protein gp130 [235], while an alternative “trans signaling” pathway also coexists [236]. The study by Moleres et al. in 2012 correlated a high GRS score, including the obesity risk allele G in the rs1800795 (−174 G > C) SNP of the *IL6* gene, with greater BMI and fat mass at the beginning, as well as with greater BMI and fat mass reduction following the introduction of lifestyle interventions [61]. Interleukin 6 (IL6) is expressed in multiple tissues, including the hypothalamus, and serves a dual role as a proinflammatory cytokine and anti-inflammatory myokine, explaining its pleiotropic functions [38]. Beyond its crucial role in immunology, infection, and inflammation, IL6 is also a homeostatic regulator implicated in energy, glucose, protein, and lipid metabolism [237]. In humans, variations in the IL6 gene have been associated with BMI and other measures of adiposity, such as waist circumference [38,238,239]. The beneficial effect of IL6 on metabolism regulation is supported by animal models in which exogenous IL6 administration led to weight reduction and by treatment of patients with Castleman disease with anti-IL6 antibody receptor, which reversed disease-associated cachexia [237,240]. In patients with obesity, IL6 is overproduced by white adipose tissue [240]. Increased IL6 levels found in obesity possibly serve as an adaptive mechanism in order to limit inflammation and balance metabolic comorbidities [236]. In obesity, stress, hormones (insulin, catecholamines, glucocorticoids), hypoxia, and inflammation interact and affect IL6 production [240]. As mentioned above, obesity results in a low-grade chronic systemic inflammation associated with increases in two inflammatory mediators, IL-6 and TNF-α [39]. Regarding the *IL6* (rs1800795) polymorphism, some studies suggest that the obesity risk allele is the G, while others suggest that it is the C [241,242]. Moreover, carriers of the C allele in rs1800795 show increased postprandial fat oxidation [243], and this allele protects from weight regain after weight loss alongside the G allele in rs1801282 of *PPARγ2* [244]. During physical exercise, IL6 produced from skeletal muscles is upregulated up to 100-fold, independently of TNF-α and without a significant increase in other inflammatory molecules, leading to insulin sensitivity, lipolysis, and fat oxidation [38,245]. The increase in IL6 expression during exercise also suppresses hyperphagia and reduces obesity-induced hypothalamic inflammation, thereby promoting insulin and leptin sensitivity and re-establishing balances in the control of appetite and energy homeostasis [246].

In summary, in the present study, we systematically reviewed the literature, aiming to decode the interaction of the genotype with diet, physical activity, and behavior interventions in children and adolescents with overweight and obesity. We found statistically significant associations with variants in 24 genetic loci, exercising an important effect on BMI and/or body composition change through lifestyle interventions in pediatric subjects. Such knowledge will enable us to overcome the present limitations of obesity lifestyle interventions and design targeted and personalized interventions to prevent and manage obesity during childhood based on individual genotype–nutrition and genotype–exercise interactions early in life. In the future, a knowledge of the individual genotype alongside a thorough understanding of the pathophysiological mechanisms mediating obesity and gene–environment interactions may contribute significantly to the prevention or management of excess adiposity in individuals that are genetically predisposed to and/or with overweight and obesity. Considering the comorbidities of obesity early in life, from the age of childhood and adolescence, as well as the independent risk of developing such obesity-induced diseases later in life, it becomes evident that such interventions should be implemented from a young age.

Our review has several important strengths. The methodology was structured on a strict application of the PRISMA guidelines. To the best of our knowledge, this is the first review to systematically examine the gene–environment interplay in childhood obesity lifestyle interventions, and it also examined the potential underlying pathophysiological mechanisms. As far as the limitations are concerned, most of the included studies focused on genetic variations on a limited number of genes, from one to three, with few of them having examined a greater number [62,69], reaching a maximum in studied variants of 56 obesity susceptibility loci in the study by Heitkamp et al. [55]. The cumulative effect of the genetic loci was examined only by Moleres et al. in 2012 [61] and Hollensted et al. [56], who used a genetic risk score approach. In addition, a great heterogeneity in the duration of the interventions was noted, ranging from 4 weeks to 10 years. This subject has long been debated in the literature, with the necessity for continuous weight loss and maintenance interventions [247,248]. A recent systematic review concerning the pediatric population suggested a minimum intervention of 6 months to reach a reduction in BMI/BMI z-score [76]. Other limitations to consider are the small sample size in many of the studies, the heterogeneity in the components of the interventions, and the lack of studies examining the effect of CNVs.

## 5. Conclusions

Preventing and treating obesity early in life, in childhood, and in adolescence should be a priority for public healthcare systems. Despite advances in pharmacotherapy such as the approval of liraglutide, a glucagon-like peptide (GLP-1) analogue, and setmelanotide, an MC4R agonist, for use in the pediatric population and the improvement in the techniques of bariatric surgery towards the management of obesity during adolescence, multidisciplinary lifestyle intervention programs remain the first-line treatment. Behavioral management programs have been proven successful in the improvement of BMI, body composition, and/or cardiometabolic profile in children and adolescents with overweight and obesity. Nevertheless, an inter-individual variability in the response to such interventions is observed, and a remission often occurs after the end of the active phase of interventions, leading to moderate results in the long term. This variability may be partially explained by genetic variations, genetic susceptibility to obesity, and gene–environment interactions. Therefore, interest has been focused on precision medicine and the design and implementation of multidisciplinary, personalized obesity lifestyle interventions through the decoding of the genetic and molecular/cellular pathophysiology of obesity and the gene–environment interactions.

Overall, it is of paramount importance to design robust GWAS, using either genome-wide SNP micro-arrays or whole-genome sequencing (WGS) techniques, in which a large sample of pediatric patients with overweight and obesity undergo structured obesity management lifestyle interventions of adequate duration. Such studies would enable researchers to study a vast number of genetic loci throughout the genome, providing an integrated view, further elucidating the gene–environment interplay, and facilitating translational research.

## Figures and Tables

**Table 1:** PICO Model.

Population	Overweight and Obese Children and Adolescents
Intervention	Obesity lifestyle management interventions
Comparison	Carriers of at-risk genotypes (SNPs or CNVs) for obesity versus non-carriers of at-risk genotypes for obesity
Outcome	Change of BMI or other measures of body composition

PICO: P—Populations/People/Patient/Problem, I—Intervention(s), C—Comparison, O—Outcome, SNPs: single-nucleotide polymorphisms, CNVs: copy number variants, BMI: body mass index.

**Table 2:** Inclusion and Exclusion Criteria.

Inclusion Criteria	Exclusion Criteria
Age ≤ 20 years	Age > 20 years
Any language	In vitro or animal studies
Any geographic location	Reviews, editorials, books and book chapters, notes, letters, conference papers, surveys
Any publication dates	Preventive intervention programs for obesity development
Species: Humans	Monogenic and/or syndromic obesity
Obesity lifestyle interventional studies	Investigation for other mutations except SNPs or CNVs
BMI: overweight/obesity	Studies examining ΒΜΙ and/or body composition change at a time after the end of a lifestyle intervention program
Common/polygenic obesity	Pharmacological or bariatric surgery obesity management interventions
Outcomes examined: change in ΒΜΙ and/or body composition in relation to the genotype	Other outcomes examined and not ΒΜΙ and/or body composition change (e.g., gene expression)
Examining the effect of SNPs and/or CNVs	

SNPs: single-nucleotide polymorphisms, CNVs: copy number variants, BMI: body mass index.

**Table 3:** General characteristics of the interventional studies included in the systematic review.

References	Study Type	Sample Size, N	Country	Age (Years), Mean Age ± SD or Range	Female (%)	Pubertal Status	Obesity Definition
Barbian et al., J. Pediatr. Genet., 2019 [48]	Quasi-Experimental	34	Brazil	10–15	73.5%	Prepubertal: 41.1% Continuous Maturation: 50% Matured: 8.9%	WHO
Corgosinho et al., Neuropeptides, 2017 [49]	Cohort	76	Brazil	15–19	N/A	100% Post-Pubertal	CDC
Deram et al., J. Clin. Endocrinol. Metab., 2008 [50]	Cohort	335	Brazil	10.7 ± 1.3	66.6%	49% Pubescent	CDC
do Nascimento et al., Eur. J. Nutr., 2017 [51]	Longitudinal	Children: 136 OW/OB, 172 NW Women: 126	Brazil	Children: 8–17, 13.55 ± 2	33.7%	N/A	WHO
Gajewska et al., Nutrients, 2016 [52]	Cohort	100	Poland	5–10	53%	100% Prepubertal	Polish Ref. Chart
Gao et al., Exp. Physiol., 2015 [53]	Cohort	55	China	16.55 ± 2.87	49%	N/A	Chinese Ref. Chart
Hagman et al., Pediatr. Diabetes, 2018 [54]	Cohort	434 (Overall) 214 (*FTO* genotyping)	USA	4–20, 12.4 ± 2.7	64.5%	31.3% Prepubertal	CDC
Heitkamp et al., JAMA Pediatr., 2020 [55]	Cohort	1198	Germany	14.0 ± 2.2	56%	N/A	IOTF
Hollensted et al., Obesity, 2018 [56]	Baseline: Case Control Follow-Up: Cohort	Baseline: Cases: 920 OW/OB Controls: 698 NW Follow-Up: 754	Denmark	Cases: 11.63 (9.59–13.87) Controls: 12.50 (10.09–15.10)	57.7%	N/A	Danish Ref. Chart
Holzapfel et al., Eur. J. Endocrinol., 2011 [57]	Cohort	310	Germany	8–19, 14 ± 2	60.3%	N/A	German Ref. Chart
Knoll et al., Horm. Metab. Res., 2012 [58]	Cohort	453	Germany	10.8 ± 2.6	55%	N/A	German Ref. Chart
Lai et al., Int. J. Biol. Sci, 2013 [59]	Cohort	88	China	14.11 ± 3.63	50%	N/A	N/A
Leite et al., Mortiz, 2017 [60]	Randomized Control Trial	47	Brazil	12–16, 15.05 ± 1.07	44.6%	Tanner Stage: 4 or 5	WHO
Moleres et al., J. Pediatr., 2012 [61]	Cohort	168	Spain	12–16, 14.6 ± 0.09	62%	N/A	IOTF
Moleres et al., Nutr. Hosp., 2014 [62]	Cohort	199	Spain	12–16, 14.5 ± 0.08	61%	N/A	IOTF
Moraes et al., An. Acad. Bras. Cienc., 2016 [63]	Cohort	36 Control Group: 17 Intervention Group: 19	Brazil	8–16 Control Group: 11.3 ± 1.6 Ιnterventiοn Group: 10.2 ± 2.2	58.3%	N/A	CDC
Müller et al., BMC Med. Genet., 2008 [64]	Baseline: Case Control Follow-Up: Cohort	Baseline: Cases: 519 Children Controls: 178 Adults Follow up: 207	Germany	Follow-Up: 10.79 ± 2.52	Follow up: 54.5%	N/A	IOTF
Reinehr et al., Diabetes, 2008 [65]	Cohort	293	Germany	6–16, 10.8 ± 2.7	55%	51% Prepubertal, 30% Pubertal, 19% Post-Pubertal	IOTF
Reinehr et al., Arch. Dis. Child., 2009 [66]	Cohort	280	Germany	10.8 (4.5–16.5)	45%	N/A	German Ref. Chart
Roth et al., BMC Pediatr., 2013 [67]	Βaseline: Case Control Follow-Up: Longitudinal	451 (28 OW, 423 OB) 583 Lean Adults	Germany	Children: 12.0 (10.0–13.7) Adults: 25.3 (22.5–26.8)	54.9%	N/A	IOTF
Santoro et al., Am. J. Clin. Nutr., 2007 [68]	Baseline: Case Control Follow-Up: Cohort	184 OB 100 Non-OΒ Controls	Italy	9.2 ± 2	41.8%	82% Prepubertal	Italian Ref. Chart
Scherag et al., Obesity, 2011 [69]	Longitudinal Cohort	401 Children 626 Adults	Germany	10.74 ± 2.55	54.6%	53.9% Prepubertal	IOTF
Schum et al., Exp. Clin. Endocrinol. Diabetes, 2012 [70]	Longitudinal	75	Germany	12.6 ± 2.6	46.6%	Pubertal: Heterozygous: 55.8% Homozygous: 76.2%	N/A
Vogel et al., Obes. Facts, 2011 [71]	Baseline: Case Control Follow-Up: Cohort	Baseline Cases: 889 Controls: 442 Follow-Up: 367	Germany	Baseline: Cases: 10.69 ± 2.98 Controls: 18.31 ± 1.10 Follow-Up: 10.77 ± 2.66	Baseline: Cases: 53.2% Controls: 61.3% Follow-Up: 55.9%	N/A	IOTF
Volckmar et al., Exp. Clin. Endocrinol. Diabetes, 2013 [72]	Baseline: Case Control Follow-Up: Cohort	Baseline: Cases: 454 Controls: 435 Follow-Up: 454	Germany	6–16, 10.8 ± 2.6	55%	N/A	German Ref. Chart
Zlatohlavek et al., Clin. Biochem., 2013 [73]	Cohort	357	Czech Republic	8–15, 13.7 ± 4.9	61%	N/A	N/A
Zlatohlavek et al., Med. Sci. Monit., 2018 [74]	Cohort	684	Czech Republic	12.7 ± 2.1	59%	N/A	N/A

Abbreviations: NW: normal weight, OW: overweight, OB: obese, N/A: not available, BMI: body mass index, SDS: standard deviation score, SNP: single-nucleotide polymorphism, GRS: genetic risk score, WHO: World Health Organization, CDC: Centers for Disease Control and Prevention, IOTF: International Obesity Task Force.

**Table 4:** Summary of the main associations identified between SNPs and BMI and/or body composition change after multidisciplinary lifestyle interventions in children and adolescents with excess adiposity.

References	Genes	Main Findings
	**Central nervous system and obesity**	
Heitkamp et al., JAMA Pediatr., 2020 [55]	*CADM2*	The G allele in rs13078960 SNP is associated with decreased BMI-SDS reduction
Roth et al., BMC Pediatr., 2013 [67]	*DRD2*	Homozygosity for the Τ allele in rs18000497 SNP is associated with decreased BMI-SDS reduction
Heitkamp et al., JAMA Pediatr., 2020 [55]	*LMX1B*	Homozygosity for the A allele in rs10733682 SNP is associated with greater weight loss
Hollensted et al., Obesity, 2018 [56]		The T allele in rs3829849 SNP is correlated with decreased BMI-SDS reduction
Gajewska et al., Nutrients, 2016 [52]	*LEPR*	Carrying at least one minor G allele in Q223R together with the wild-type K665N is associated with the greatest weight loss and fat mass reduction
Corgosinho et al., Neuropeptides, 2017 [49]		Homozygosity for the T allele in rs2767485 SNP is associated with greater BMI reduction
Zlatohlavek et al., Clin. Biochem., 2013 [73]	*MC4R*	Homozygosity for the C allele in s17782313 SNP is associated with greater weight loss outcomes
Moleres et al., J. Pediatr., 2012 [61]		The C allele in s17782313 SNP is associated with greater BMI and body composition reduction as part of a GRS
Vogel et al., Obes. Facts, 2011 [71]		The C allele in rs17782313 SNP or the A allele in rs12970134 SNP in females are associated with more efficient BMI-SDS reduction than in males
Santoro et al., Am. J. Clin. Nutr., 2007 [68]	*MC3R*	The 6Lys allele of rs3746619 and the 81Ile allele of rs3827103 are associated with reduced BMI change
	**Adipose tissue and obesity**	
Heitkamp et al., JAMA Pediatr., 2020 [55]	*RPTOR*	Homozygosity for the G allele in the rs12940622 SNP is associated with reduced weight loss
Barbian et al. [48], do Nascimento et al. [51], Müller et al. [64], Moraes et al. [63], Hollensted et al. [56], Scherag et al. [69], Schum et al. [70]	*FTO*	Τhe rs9939609, rs1421085, rs1558902, rs1421085, rs17817449, rs9939609 SNPs are not associated with BMI or body composition change
Moleres et al., J. Pediatr., 2012 [61]		The A allele in rs9939609 SNP is associated with greater BMI reduction both individually and as a part of a GRS
Reinehr et al., Arch. Dis. Child., 2009 [66]		Homozygosity for the A allele in rs9939609 SNP of *FTO* together with homozygosity for the C allele in rs7566605 SNP of *INSIG2* is associated with decreased BMI reduction
Zlatohlavek et al., Clin. Biochem., 2013 [73]		Homozygosity for the G allele in rs17817449 SNP of *FTO* is associated with greater BMI reduction both individually and in synergy with homozygosity for the C allele in rs17782313 SNP of *MC4R*
Hagman et al., Pediatr. Diabetes, 2018 [54]		Homozygosity for the A allele in rs8050136 SNP is correlated with greater BMI reduction
Heitkamp et al., JAMA Pediatr., 2020 [55]	*ETS2*	Homozygosity for the C allele in rs2836754 SNP is associated with greater body weight and BMI reduction
Heitkamp et al., JAMA Pediatr., 2020 [55]	*KAT8*	The A allele in rs9925964 SNP is associated with greater BMI-SDS reduction
Moleres et al., J. Pediatr., 2012 [61]	*TMEM1*	The G allele in rs7561317 SNP is associated with greater BMI-SDS and fat mass reduction individually and as a part of a GRS
Hollensted et al., Obesity, 2018 [56], Scherag et al., Obesity, 2011 [69], Zlatohlavek et al., Med. Sci. Monit., 2018 [74]		The rs4854349, rs4854344, and rs11127485 SNPs are not associated with BMI or body composition change
Moleres et al., J. Pediatr., 2012 [61]	*PPARγ*	The G allele of rs1801282 SNP is associated with a greater BMI and fat mass reduction as part of a GRS
Scherag et al., Obesity, 2011 [69]	*SDCCAG8*	Homozygosity for the T allele in rs10926984 SNP, the T allele in rs12145833 SNP and the C allele in rs2783963 SNP is associated with reduced BMI change
Heitkamp et al., JAMA Pediatr., 2020 [55]	*CPNE8*	The A allele in rs11170468 SNP is associated with resistance to BMI-SDS reduction
	**Adipose tissue metabolism**	
Moleres et al., Nutr. Hosp., 2014 [62]	*APOA1*	The A allele in rs670 SNP is associated with greater weight and BMI reduction, while combined analyses with the A allele in rs1800777 SNP explains up to 24% of BMI-SDS amelioration
Moleres et al., Nutr. Hosp., 2014 [62]	*CETP*	The A allele in rs1800777 is associated with greater weight and BMI reduction, while combined analyses with the A allele in rs670 SNP explains up to 24% of BMI-SDS amelioration
Gao et al., Exp. Physiolm., 2015 [53]	*LPL*	Homozygosity for the G allele in rs283 SNP is associated with greater body fat reduction
Reinehr et al., Diabetes, 2008 [65], Reinehr et al., Arch. Dis. Child., 2009 [66]	*INSIG2*	Homozygosity for the C alle in rs7566605 SNP is associated with lower BMI and BMI-SDS reduction
Reinehr et al., Arch. Dis. Child., 2009 [66]		Homozygosity for the C allele in rs7566605 SNP of *INSIG2* together with homozygosity for the A allele in rs9939609 SNP of *FTO* is associated with decreased BMI reduction
Deram et al., J. Clin. Endocrinol. Metab., 2008 [50]	*PLIN1*	The T allele in rs1052700 is associated with greater BMI and body composition change
Moleres et al., J. Pediatr., 2012 [61]	*ADIPOQ*	The C allele in rs822395, the G allele in rs2241766, and the T allele in rs1501299 SNPs are associated with a greater BMI and fat mass reduction as part of a GRS
Gajewska et al., 2016, Nutrients [52]		The rs266729 and rs1686119 SNPs are not associated with BMI or body composition change
	**Adipose tissue inflammation and obesity**	
Heitkamp et al., JAMA Pediatr., 2020 [55]	*IFNGR1*	Homozygosity for the G allele of the rs13201877 SNP is associated with greater weight and BMI reduction
Heitkamp et al., JAMA Pediatr., 2020 [55]	*SLC39A8*	The T allele in rs13107325 SNP is associated with decreased BMI-SDS reduction
Moleres et al., J. Pediatr., 2012 [61]	*IL6*	The G allele in rs1800795 SNP is associated with a greater BMI and fat mass reduction as part of a GRS

Abbreviations: ΒΜΙ: body mass index, SNP: single-nucleotide polymorphism, GRS: genetic risk score.

## Data Availability

Not applicable.

## Supplementary Materials

The following supporting information can be downloaded at https://www.mdpi.com/article/10.3390/nu15061416/s1: Table S1: Lifestyle intervention characteristics; Table S2: Newcastle—Ottawa Quality Assessment Scale for Non-Randomized Control Studies—Cohort Studies; Table S3: Newcastle—Ottawa Quality Assessment Scale for Randomized Controlled Trials; Table S4: BMI and body composition outcomes in relation to the genotype.
