# Supplementary material for: The Genetic Basis of Childhood Obesity: A Systematic Review
Source: Nutrients. 2023 Mar 15;15(6):1416. doi: 10.3390/nu15061416 (PMC10058966; doi:10.3390/nu15061416)
Supplement: Supplementary file 1 [file nutrients-15-01416-s001.zip › nutrients-2198578-supplementary.pdf]

Systematic Review

The Genetic Basis of Childhood Obesity: A Systematic Review

Supplementary Material Table S1: Lifestyle intervention characteristics

References	Type of Intervention	Duration	Dietary Intervention	Exercise	Phycological Counseling	Compliance		Drop out
						Patient Reported	Other measures of evaluation	
Barbian *et al*, J Pediatr Genet, 2020 [48]	Interdisciplinary intervention program at a campus<br>• Intervention group: physical exercises, nutritional, and psychological counseling<br>• Control group: not received any kind of intervention	6 months	Food re-education<br>1-hour, once-week	3 times per week<br>Warm up, walking, stretching, sports, aquatic activities, resisted and aerobic exercises, circuit and respiratory exercises	Once a week, 1 hour<br>Group intervention (cognitive orientation/techniques in the handling of obesity-related thoughts)	N/A	Participation > 70% in all sessions	None
Corgosinho *et al*, Neuropeptides, 2017 [49)]	Multicomponent weight loss therapy<br>(clinical, nutritional, and psychological counselling and exercise training)	1 year	Individual and parents` nutritional consultation<br>1 hour, once-week<br>Energy intake: set at the levels	3 times-week<br>30 min of aerobic training + 30 min of resistance training	Group sessions<br>Psychodynamic approach<br>1/week	N/A	N/A	None

			recommended by the dietary reference intake for subjects with low levels of physical activity of the same age and sex, following a balanced diet					
Deram *et al*, J Clin Endocrinol Metab, 2008 [50]	Lifestyle and Nutritional Reeducation (Nutritionists, physical therapists, psychologists, and endocrinologists)	20 weeks	Personalized nutritional orientation Monthly Average of 1800 kcal/d/no restrictive but balanced diet education program	Group meeting with a physical educator Monthly	Eating behavior group meeting with a psychologist Monthly Psychiatrist evaluation when necessary	N/A	N/A	22.4%
do Nascimento *et al*, European Journal of Nutrition, 2017 [51]	Children/adolescents: physical exercise intervention Obese women: hypocaloric dietary intervention	Children: 12 weeks Women: 7 weeks	Women: Hypocaloric diet: a deficit of 600 kcal/per Energy consumption (fats: 20-35%, carbohydrates: 45-65%, proteins: 10-35%) Dietary reeducation	Children: aerobic exercise, high-intensity interval training, combined training, water walking Conducted by physical	N/A	N/A	N/A	Women: 36.6%

				education professionals<br>3 times/week in home schools				
Gajewska *et al*, Nutrients, 2016 [52]	Outpatient Multidisciplinary Intervention Program	3 months	A low-energy diet for children and their parents<br>Recommended daily energy intake: 1200–1400 kcal/day<br>Diet composed: 20% protein, 30% fat, 50% carbohydrates<br>3–5 meals every day	Physical activity instructions<br>Reduction of sedentary activities (< 2 hours/day)	Individual psychological care for the child and his/her family	N/A	Daily energy intake and percentage of dietary reference intake: similar in children with and without weight loss	24%
Gao *et al*, Exp Physiol, 2015 [53]	Exercise intervention in weight-reducing summer camp<br>(Exercise training with medical supervision and dietary control)	4 weeks	Daily energy intake per person (in kilocalories per day) = ideal weight × energy intake during light physical activity (25 kcal kg^{-1})<br>Energy supply: Sugar 60-70%, fat 10-	7 days per week, twice a day, for 120 min each session<br>Cycling, jogging, step-aerobics and brisk walking, including strength training three	N/A	N/A	N/A	None

			15%, Protein 20-25%<br>Energy supply proportions: Breakfast 30%, Lunch: 40%, Supper 30%	times per week<br>Heart rate monitor (heart rate +20–40% heart rate reserve)				
Hagman *et al*, Pediatr Diabetes, 2018 [54]	Clinic based behavioral modification therapy aimed at child and parents<br>(Diet, exercise and psychological, social worker counseling and medical evaluation biannually)	35.9 ±20.8 months<br>(12 months to 10 years)	General diet counselling by a dietician<br>Biannually	Exercise counseling by sports<br>therapist<br>Biannually	Brief psychosocial counseling<br>Biannually	N/A	Poor response to the intervention:<br>≥0.14 BMI Z-score units change<br>Positive response:<br>≤ −0.22 Z-score units change	N/A
Heitkamp *et al*, JAMA Pediatr, 2020 [55]	In-Hospital Lifestyle Intervention Program	4 to 6 weeks	Calorie-restricted diet<br>(30% fat, 15% proteins and 55% carbohydrates of the total energy content)<br>Energy intake: 1,250 - 1,800 kcal/day,	10 h/week: organised physical activity<br>6 hours/week: recreational exercise	Theoretical and practical lessons on healthy eating, physical activity and behavior change skills based on the cognitive-behavioral theory<br>16 sessions within 4 weeks	N/A	N/A	N/A

			depending on height and sex					
Hollensted *et al*, Obesity, 2018 [56]	Family-centered – Multidisciplinary Behavioral Lifestyle Intervention<br>(Evaluation and counselling by a team of pediatricians, pediatric nurses, dieticians, psychologists and/or social workers)	6 to 24 months<br>Median<br>1.25 (1.00 to 1.59) years	Dietician counselling:<br>one hour initially, 30 min thereafter at individual intervals	Physical activity recommende d changes	Psychologist and/or a social worker support	N/A	Participation at appointment s<br>BMI-SDS decrease	N/A
Holzapfel *et al*, European Journal of Endocrinology, 2011 [57]	In Patient Lifestyle Intervention Program/<br>¨Long-term effects of a lifestyle<br>intervention in obesity and genetic influence in children¨ - (LOGIC) study	4-6 weeks	Moderate energy reduction: -500 kcal/day	Physical activities such as swimming and walking (11 h/week)	Behavior therapy	N/A	N/A	4 weeks: 4.1%<br>6 weeks: 39.6%
Knoll *et al*, Horm Metab Res 2012 [58]	An Outpatient Lifestyle Intervention Program/<br>"Obeldicks<br>(Exercise, dietary, behavioral intervention, and medical evaluation)	1 year	'Optimised Mixed Diet'<br>Fat and Sugar Reduction<br>30% Fat, 15% Proteins, 55% Carbohydrates , 5% Sugar	Exercise sessions: Once/week	Behavior therapy: contingency contracting, self-monitoring,<br>praise, stimulus control techniques, problem-solving	N/A	Participation on therapy trials and exercise groups, Changes in weight status	None

Lai *et al*, Int. J. Biol. Sci, 2013 [59]	Aerobic exercise training program<br>(Exercise intervention with dietary adaptation and medical evaluation)	4 weeks	A two-day unified dietary adaptation	Customized aerobic training<br>120-min training sessions, 5 days/week<br>(warm-up, aerobic exercise at an intensity of 20-40% of heart rate reserve and a cool down)	N/A	N/A	N/A	None
Leite *et al*, Mortiz, 2017 [60]	Aerobic and resistance training program<br>(multidisciplinary team assessment)	12 weeks	N/A	Resistance and aerobic training<br>Three times/week	N/A	N/A	Participation > 70% of the training sessions	N/A
Moleres *et al*, J Pediatr., 2012 [61]	A Multidisciplinary Lifestyle Intervention/<br>The EVASYON Study<br>(nutritionists, physical therapists, psychologists, and pediatricians)	3 months	Nutritional advice<br>Weekly group session<br>Total energy: 30% fat, 15% proteins, 55% carbohydrates	Physical activity program<br>Weekly group sessions<br>Calculated baseline metabolism and computed an	Psychological support<br>Weekly group session	N/A	N/A	N/A

				activity metabolic equivalent index (metabolic equivalent tasks-h/week), which represents the physical exercise during the week for each participant.				
Moleres *et al*, Nutricion Hospitalaria, 2014 [62]	A Multidisciplinary Lifestyle Intervention/ The EVASYON Study (nutritionists, physiotherapists, psychologists, paediatricians)	10 weeks	Nutritional advice Weekly group session Total energy: 30% fat, 15% proteins, 55% carbohydrates	Physical activity program Weekly group sessions Calculated baseline metabolism and computed an activity metabolic equivalent index (metabolic equivalent tasks-h/ week), which represents the	Psychological support Weekly group session	N/A	N/A	N/A

				physical exercise during the week for each participant.				
Moraes *et al,* An Acad Bras Cienc, 2016 [63]	Multidisciplinary Lifestyle Intervention (physical education professional, physiotherapist, pharmacist, nutritionist, psychologist, doctor and dentist)	4 months	Nutrition counseling (15 minutes) (educational lectures, theater productions, folders, healthy cooking classes, movie sessions, educational games and the deployment of a fruitful garden)	Warmup (10 minutes), aerobic exercises (50 minutes), exercises with entertaining features (20 minutes)	Postural re-education and oral health counseling.	N/A	N/A	None
Müller *et al*, BMC Medical Genetics 2008 [64]	An outpatient intervention program/ "Obeldicks" (paediatricians, dietitians, psychologists and exercise physiologists)	1 year	Optimised mixed diet Fat and sugar reduced diet compared with the everyday nutrition of German children Energy: 30% fat, 15% proteins, 55%	Aerobic exercise, lifestyle exercise and decreasing sedentary behavior Once weekly	Contingency contracting, self-monitoring, praise, stimulus control techniques and problem-solving	Questionnaire concerning eating and exercise habits	Participation in exercise groups	None

			carbohydrates including 5% sugar					
Reinehr *et al*, Diabetes, 2008 [65]	Outpatient intervention program/"Obeldicks" (paediatricians, dietitians, psychologists and exercise physiologists)	1 year	Optimised mixed diet<br>Fat and sugar reduced diet compared with the everyday nutrition of German children<br>Energy: 30% fat, 15% proteins, 55% carbohydrates including 5% sugar	Aerobic exercise, lifestyle exercise and decreasing sedentary behavior<br>Once weekly	Contingency contracting, self-monitoring, praise, stimulus control techniques and problem-solving	Questionnaire concerning eating and exercise habits	Participation in exercise groups	Motivation phase: 26%<br>First 3 months of the Intervention: 11%
Reinehr *et al*, Arch Dis Child, 2009 [66]	Outpatient intervention program/<br>"Obeldicks"<br>(paediatricians, dietitians, psychologists<br>and exercise physiologists)	1 year	Optimised mixed diet<br>Fat and sugar reduced diet compared with the everyday nutrition of German children<br>Energy: 30% fat, 15% proteins, 55% carbohydrates	Aerobic exercise, lifestyle exercise and decreasing sedentary behavior<br>Once weekly	Contingency contracting, self-monitoring, praise, stimulus control techniques and problem-solving	Questionnaire concerning eating and exercise habits	Participation in exercise groups	14%

			including 5% sugar					
Roth *et al*, BMC Pediatrics, 2013 [67]	Outpatient intervention program/ “Obeldicks” (paediatricians, dietitians, psychologists and exercise physiologists)	1 year	Optimised mixed diet<br>Fat and sugar reduced diet compared with the everyday nutrition of German children<br>Energy: 30% fat, 15% proteins, 55% carbohydrates including 5% sugar	Aerobic exercise, lifestyle exercise and decreasing sedentary behavior<br>Once weekly	Contingency contracting, self-monitoring, self-praise, stimulus control techniques and problem-solving	Questionnaire concerning eating and exercise habits	Participation in exercise groups	None
Santoro *et al*, Am J Clin Nutr, 2007 [68]	Weight Reduction Program	12 months	A nutritionally balanced diet<br>Energy: 50% carbohydrate, 30% fat, 20% protein<br>Self-selected diet of common foods: 60% of the recommended dietary energy allowances for age and sex	Physical exercise	Individual psychological care of the child and his or her family	Level of habitual physical activity, expressed as a score, based on a standardized questionnaire	N/A	446/630 children dropped out (not included in the study)

Scherag *et al*, Obesity, 2011 [69]	Children: Outpatient lifestyle intervention program/"Obeldicks" (paediatricians, dietitians, psychologists and exercise physiologists) Adults*:* Hypoenergetic diet	Children: 1 year Adults: 10 weeks	Children: Optimised mixed diet Fat and sugar reduced diet compared with the everyday nutrition of German children Energy: 30% fat, 15% proteins, 55% carbohydrates including 5% sugar Adults: Hypoenergetic diet (NUGENOB)	Children*:* Aerobic exercise, lifestyle exercise and decreasing sedentary behavior Once weekly	Children*:* Contingency contracting, self-monitoring, praise, stimulus control techniques and problem-solving	Questionnaire concerning eating and exercise habits	Participation in exercise groups	None
Schum *et al*, Exp Clin Endocrinol Diabetes, 2012 [70]	Lifestyle intervention program (close surveillance by one physician)	6 months	Optimized mixed diet	Increase from 1 to 2 hours per day, including all daily life activities and fitness training	N/A	N/A	N/A	N/A
Vogel *et al*, Obes Facts, 2011 [71]	A lifestyle intervention program/ "Obeldicks"	1 year	Optimised mixed diet Fat and sugar reduced diet compared	Aerobic exercise, lifestyle exercise and decreasing	Contingency contracting, self-monitoring, praise, stimulus control	Questionnaire concerning eating and exercise habits	Participation in exercise groups	None

	(paediatricians, dietitians, psychologists and exercise physiologists)		with the everyday nutrition of German children<br>Energy: 30% fat, 15% proteins, 55% carbohydrates including 5% sugar	sedentary behavior<br>Once weekly	techniques and problem-solving			
Volckmar *et al*, Exp Clin Endocrinol Diabetes, 2013 [72]	A lifestyle intervention program/ "Obeldicks"<br>(paediatricians, dietitians, psychologists and exercise physiologists)	1 year	Optimised mixed diet<br>Fat and sugar reduced diet compared with the everyday nutrition of German children<br>Energy: 30% fat, 15% proteins, 55% carbohydrates including 5% sugar	Aerobic exercise, lifestyle exercise and decreasing sedentary behavior<br>Once weekly	Contingency contracting, self-monitoring, praise, stimulus control techniques and problem-solving	Questionnaire concerning eating and exercise habits	Participation in exercise groups	None
Zlatohlavek *et al*, Clinical Biochemistry,2013 [73]	Inpatient weight reduction program<br>(dietician, exercise specialist)	4 weeks	Dietary changes<br>A caloric intake of 5000 kJ for the age category of 8 to 10 years old and 7000 kJ for	Five units (50 min each) of daily, supervised physical activity, totaling at least 120	N/A	N/A	Participation in sessions	3.9%

			those 11 to 15 years old.	minutes of endurance training (heart rate of 65–75% of the maximum)<br>Aerobic and resistance training (ball games, swimming, dancing, fast walking)				
Zlatohlavek *et al*, Med Sci Monit, 2018 [74]	Inpatient weight reduction program<br>(dietician, exercise specialist)	4 weeks	Dietary changes<br>A caloric intake of 5000 kJ for the age category of 8 to 10 years old and 7000 kJ for those 11 to 15 years old.	Five units (50 min each) of daily, supervised physical activity, totaling at least 120 minutes of endurance training (heart rate of 65–75% of the maximum)<br>Aerobic and resistance training (ball games, swimming, dancing, fast walking)	N/A	N/A	Participation in sessions	None

Abbreviations: N/A: not available

Supplementary Material Table S2: Newcastle – Ottawa Quality Assessment Scale for Non-Randomized control studies - Cohort studies

References	Selection				Comparability	Outcome			Quality Score
	Representativeness of Exposed Cohort	Selection of the Non-Exposed Cohort from same source as exposed cohort	Ascertainment of Exposure	Outcome of Interest Was Not Present at Start of Study	Comparability of Cohorts (*Age/Sex,*Other Factors)	Assessment of Outcome	Follow-Up Long Enough for Outcome to Occur (*≥6 months)	Adequacy of Follow-Up	
Barbian *et al*, J Pediatr Genet, 2019 [48]	*	*	*	*	**	*	*	*	Good
Corgosinho *et al*, Neuropeptides, 2017 [49]	*	*	*	*	**	*	*	*	Good
Deram *et al*, J Clin Endocrinol Metab, 2008 [50]	*	*	*	*	**	*	-	*	Good
do Nascimento *et al*, European Journal of Nutrition, 2017 [51]	*	*	*	*	*	*	-	*	Good
Gajewska *et al*, Nutrients, 2016 [52]	*	*	*	*	**	*	-	*	Good
Gao *et al*, Exp Physiolm, 2015 [53]	*	*	*	*	*	*	-	*	Good
Hagman *et al*, Pediatr Diabetes, 2018 [54]	*	*	*	*	**	*	*	*	Good
Heitkamp *et al*, JAMA Pediatr,	*	*	*	*	*	*	-	*	Good

2020 [55]									
Hollensted *et al*, Obesity, 2018 [56]	*	*	*	*	**	*	*	*	Good
Holzapfel *et al*, European Journal of Endocrinology, 2011 [57]	*	*	*	*	*	*	-	*	Good
Knoll *et al*, Horm Metab Res 2012 [58]	*	*	*	*	**	*	*	*	Good
Lai *et al*, Int. J. Biol. Sci, 2013 [59]	*	*	*	*	**	*	-	*	Good
Moleres *et al*, J Pediatr, 2012 [61]	*	*	*	*	*	*	-	*	Good
Moleres *et al*, Nutricion Hospitalaria, 2014 [62]	*	*	*	*	*	*	-	*	Good
Moraes *et al*, An Acad Bras Cienc, 2016 [63]	*	*	*	*	*	*	-	*	Good
Müller *et al*, BMC Medical Genetics 2008 [64]	*	*	*	*	*	*	*	-	Good
Reinehr *et al*, Diabetes, 2008 [65]	*	*	*	*	*	*	*	*	Good
Reinehr *et al*, Arch Dis Child, 2009 [66]	*	*	*	*	**	*	*	*	Good
Roth *et al*, BMC Pediatrics, 2013 [67]	*	*	*	*	**	*	*	*	Good

Santoro *et al*, Am J Clin Nutr, 2007 [68]	*	*	*	*	*	*	*	*	Good
Scherag *et al*, Obesity, 2011 [69]	*	*	*	*	**	*	*	*	Good
Schum *et al*, Exp Clin Endocrinol Diabetes, 2012 [70]	*	*	*	*	**	*	*	-	Good
Vogel *et al*, Obes Facts, 2011 [71]	*	*	*	*	**	*	*	*	Good
Volckmar *et al*, Exp Clin Endocrinol Diabetes, 2013 [72]	*	*	*	*	**	*	*	*	Good
Zlatohlavek *et al*, Clinical Biochemistry, 2013 [73]	*	*	*	*	**	*	-	*	Good
Zlatohlavek *et al*, Med Sci Monit, 2018 [74]	*	*	*	*	**	*	-	*	Good

*Asterisk: fulfillment of the criteria within the subsection, – Dash: the criteria within the subsection are not met. (A good quality score requires 3 or 4 stars in selection, 1 or 2 stars in comparability and 2 or 3 stars in outcome/exposure domain. When 2 stars are given in selection domain, 1 or 2 stars in comparability domain and 2 or 3 stars in outcome/exposure domain, fair quality is met. Finally, a poor-quality study is given 0 or 1 star in selection or 0 stars in comparability or 0 or 1 stars in outcome/exposure domain.)

Supplementary Material Table S3: Newcastle – Ottawa Quality Assessment Scale for Randomized controlled trials

**References**	**Selection**				**Comparability**	**Exposure**			**Quality Score**
	**Adequate case definition**	**Representativeness of the cases**	**Selection of Controls**	**Definition of Controls**	**Comparability of cases and controls on the basis of the design or analysis**	**Assessment of Exposure**	**Same method of ascertainment for cases and controls**	**Non-Response rate**	
Leite *et al*, Mortiz, 2017 [60]	*	*	-	*	*	*	*	*	Good

*Asterisk: fulfillment of the criteria within the subsection, – Dash: the criteria within the subsection are not met. (A good quality score requires 3 or 4 stars in selection, 1 or 2 stars in comparability and 2 or 3 stars in outcome/exposure domain. When 2 stars are given in selection domain, 1 or 2 stars in comparability domain and 2 or 3 stars in outcome/exposure domain, fair quality is met. Finally, a poor-quality study is given 0 or 1 star in selection or 0 stars in comparability or 0 or 1 stars in outcome/exposure domain.)

Supplementary Table S4. BMI and body composition outcomes in relation to the genotype.

References	Genes	SNPs or Combination of SNPs Examined	Risk/(Other) Allele/ Allelic combinations	Effect of Genotype after Intervention on:			Genetic Inheritance Model
				Weight Loss (kg)	BMI (kg/m²) / BMI-SDS Change	Body Composition Change	
Barbian *et al*, J Pediatr Genet, 2020 [48]	*FTO*	rs9939609	A (T)	N/A	-	-	Dominant
Corgosinho *et al*, Neuropeptides, 2017 [49]	*LEPR*	rs2767485	C (T)	N/A	TT carriers ↑ $P \leq 0.05$	-	Dominant
Deram *et al*, J Clin Endocrinol Metab, 2008 [50]	*PLIN1*	rs2289487	C (T)	Strong linkage disequilibrium with rs894160			Dominant
		rs894160	A (G)	-	-	-	
		rs2304795	G (A)	-	-	-	
		rs1052700	T (A)	T allele carriers ↑	T allele carriers ↑	Waist circumference in T allele carriers	

				P = 0.003	P = 0.014	↑ P = 0.05	
do Nascimento *et al*, European Journal of Nutrition, 2017 [51]	*FTO*	rs9939609	A (T)	N/A	Children: - Women: -	Children: - Women: Abdominal circumference in A allele carriers ↓ p = 0.04	Dominant
Gajewska *et al*, 2016, Nutrients [52]	*LEPR*	rs1137101 (Q223R)	G (A)	N/A	AG or GG carriers: ↑ p = 0.035	Delta Fat Mass (%) in AG or GG carriers ↑ p = 0.003	Dominant
		rs8129183 (K656N)	C (G)	N/A	-	-	
		Q223R + K656N	Q223R: AA	N/A	-	Fat Mass (%):	

			AND<br>K656N: GG			↑<br>$p < 0.001$	
			Q223R: AG or GG<br>AND<br>K656N: GG		↑<br>$p = 0.006$	Fat Mass (%):<br>↑<br>$p < 0.001$	
			Q223R: AA<br>AND<br>K656N: GC or CC		-	Fat-Free Mass (kg):<br>↑<br>$p = 0.002$	
			Q223R: AG<br>AND<br>K656N: GC		-	-	
	LEP	rs7799039	A (G)	N/A	-	-	
	ADIPOQ	rs266729	G (C)	N/A	-	-	
		rs1686119	G (A)	N/A	-	-	

Gao *et al*, 2015, Exp Physiol [53]	*LPL*	rs283	A (G)	-	N/A	Body Fat (%) in GG carriers ↑ P=0.006	Additive
Hagman *et al*, Pediatr Diabetes, 2018 [54]	*FTO*	rs8050136	A (C)	-	AA carriers vs CC: ↑ P = 0.028	-	Additive
Heitkamp *et al*, JAMA Pediatr, 2020 [55]	*TAL1*	rs977747	T (G)	-	-	N/A	Additive
	ELAVL4	rs11583200	C (T)	-	-		
	PTBP2	rs11165643	T (C)	-	-		
	GNAT2	rs17024393	C (T)	-	-		
	SEC16B	rs543874	G (A)	-	-		
	ADCY3	rs10182181	G (A)	-	-		
	KCNK3	rs11126666	A (G)	-	-		

	LRP1B	rs2121279	T (C)	-	-		
	FIGN	rs1460676	C (T)	-	-		
	UBE2E3	rs1528435	T (C)	-	-		
	CREB1	rs17203016	G (A)	-	-		
	LOC646736	rs2176040	A (G)	-	-		
	RARB	rs6804842	G (A)	-	-		
	FHIT	rs2365389	C (T)	-	-		
	CADM2	rs13078960	G (T)	-	Carriers of risk allele ↓ $P = 2.52 \times 10^{-5}$		
	ETV5	rs1516725	C (T)	-	-		
	GNPDA2	rs10938397	G(A)	-	-		

	SCARB2	rs17001654	G (C)	-	-		
	SLC39A8	rs13107325	T (C)	-	Carriers of risk allele ↓ $P = 1.67 \times 10^{-4}$		
	HHIP	rs11727676	T (C)	-	-		
	C6orf106	rs205262	G (A)	-	-		
	IFNGR1	rs13201877	G (A)	Homozygous carriers of risk allele: ↑ $P = 2.39 \times 10^{-5}$	Homozygous carriers of risk allele ↑ $P = 1.48 \times 10^{-4}$		
	PARK2	rs13191362	A (G)	-	-		
	HIP1	rs1167827	G (A)	-	-		

	HNF4G	rs17405819	T (C)	-	-		
	LINGO2	rs10968576	G (A)	-	-		
	EPB41L4B	rs6477694	C (T)	-	-		
	TLR4	rs1928295	T (C)	-	-		
	LMX1B	rs10733682	A (G)	Homozygous carriers of risk allele: ↑ P = 6.37 × 10^{-4}	-		
	GRID1	rs7899106	G (A)	-	-		
	HIF1AN	rs17094222	C (T)	-	-		
	TRIM66	rs4256980	G (C)	-	-		
	BDNF	rs11030104	A (G)	-	-		
	MTCH2	rs3817334	T (C)	-	-		

	CADM1	rs12286929	G (A)	-	-		
	CPNE8	rs11170468	A (C)	-	Carriers of risk allele ↓ P = 1.05 × 10^{-4}		
	BCDIN3D	rs7138803	A (G)	-	-		
	ZNF664-FAM101A	rs10773049	C (T)	-	-		
	OLFM4	rs12429545	A (G)	-	-		
	MIR548A2	rs1441264	A (G)	-	-		
	LOC100287559	rs7164727	T (C)	Homozygous carriers of the risk allele:	-		

				↓<br>$P = 4.02 \times 10^{-4}$			
	NLRC3	rs758747	T (C)	-	-		
	GPRC5B	rs12446632	G (A)	-	-		
	SBK1	rs2650492	A (G)	-	-		
	ATP2A1	rs3888190	A (C)	-	-		
	INO80E	rs4787491	G (A)	-	-		
	KAT8	rs9925964	A (G)	-	Carriers of risk allele<br>↑<br>$P = 3.84 \times 10^{-4}$		
	CBLN1	rs2080454	C (A)	-	-		
	RABEP1	rs1000940	G (A)	-	-		

	RPTOR	rs12940622	G (A)	Homozygous carriers of the risk allele: ↓ P = 1.86 × 10^{-5}	-		
	MC4R	rs6567160	C (T)	-	-		
	PGPEP1	rs17724992	A (G)	-	-		
	KCTD15	rs29941	G (A)	-	-		
	TOMM40	rs2075650	A (G)	-	-		
	QPCTL	rs2287019	C (T)	-	-		
	ETS2	rs2836754	C (T)	Homozygous carriers of the risk allele:	Homozygous carriers of the risk allele		

				↑ $P = 1.51 \times 10^{-4}$	↑ $P = 2.84 \times 10^{-4}$		
Hollensted *et al*, Obesity, 2018 [56]		GRS		N/A	-	N/A	Additive
	GPR61	rs7550711	T(C)		-		
	SEC16B	rs543874	G(A)		-		
	GNPDA2	rs13130484	T(C)		-		
	TFAP2B	rs987237	G(A)		-		
	FAIM2	rs7132908	A(G)		-		
	OLFM4	rs12429545	A(G)		-		
	FTO	rs1421085	C(T)		-		
	LMX1B	rs3829849	T(C)		T allele carriers ↓		

					P = 0.003		
	TMEM18	rs4854349	C(T)		-		
	MC4R	rs6567160	C(T)		-		
	RAB27B	rs8092503	G(A)		-		
	ADCY3	rs11676272	G(A)		-		
	TNNI3K	rs12041852	G(A)		-		
	ELP3	rs13253111	A(G)		-		
	ADAM23	rs13387838	A(G)		-		
Holzapfel *et al,* European Journal of Endocrinology, 2011 [57]	*MTNR1B*	rs10830963	G (C)	-	-	-	Additive
Knoll *et al*, Horm Metab Res 2012 [58]	*FAAH*	rs324420	A (C)	-	-	N/A	Dominant

Lai *et al*, Int. J. Biol. Sci, 2013 [59]	*PBEF1* (Visfatin)	rs4730153	A (G)	N/A	-	-	N/A
Leite *et al*, Mortiz, 2017 [60]	*β2 adrenergic receptor (ADRB2)*	rs1042714 (Gln27Glu)	G (C)	-	-	-	Dominant
Moleres *et al*, J Pediatr, 2012 [61]		GRS		N/A	↑ P < 0.001 (For each risk allele in the genotype, there was a 0.264 decrease in BMI-SDS)	↑ p=0.012 (For each risk allele in the genotype, there was a 0.197 decrease in fat mass percentage)	Additive
	FTO	rs9939609	A (T)		Effect allele carriers	-	

					↑ p = 0.018		
		rs7204609	C (T)		-	-	
	MC4R	rs17782313	C (T)		-	-	
	TMEM18	rs7561317	G (A)		Effect allele carriers ↑ p = 0.005	Effect allele carriers ↑ p = 0.038	
	IL6	rs1800795	G (C)		-	-	
	PPARG	rs1801282	G (C)		-	-	
	ADIPQ	rs822395	C (A)		-	-	
		rs2241766	G (T)		-	-	
		rs1501299	T (G)		-	-	
Moleres *et al*, Nutricion	*APOA1*	rs670	A (G)	Effect allele carriers	Effect allele carriers	-	N/A

Hospitalaria, 2014 [62]				↑ P = 9.8 × 10-5	↑ P < 0.001		
	APOA5	rs662799	C (T)	-	-		
	CETP	rs1800777	A (G)	Effect allele carriers ↑ P = 1.5 × 10-4	Effect allele carriers ↑ P = 0.01		
	FTO	rs9939609	A (T)	-	-		
	APOA1 +*CETP*	rs670 + rs1800777	N/A	Effect allele carriers ↑ P = 3.3 × 10-^{6}	Effect allele carriers ↑ P = 2.3 × 10-7		
Moraes *et al*, An Acad Bras Cienc, 2016 [63]	*FTO*	rs9939609	A(T)	-	-	-	Dominant
Müller *et al*,	*FTO*	rs9939609	A(T)	-	-	N/A	Additive

BMC Medical Genetics, 2008 [64]							
Reinehr *et al*, Diabetes, 2008 [65]	*INSIG2*	rs7566605	C (G)	N/A	CC carriers: ↓ P = 0.007	N/A	Recessive
Reinehr *et al*, Arch Dis Child, 2009 [66]	*INSIG2*	rs7566605	C (G)	N/A	CC carriers: ↓ $p \leq 0.001$	N/A	Additive
	FTO	rs9939609	A (T)	N/A	-	N/A	
	INSIG2 + FTO	rs7566605 + rs9939609	All possible genotype combinations	N/A	CC+AA carriers ↓ $p \leq 0.05$	N/A	
Roth *et al*, BMC Pediatrics, 2013 [67]	*DRD2*	rs18000497	T (C)	N/A	TT carriers: ↓ $p < 0.05$	-	Additive Dominant Recessive

	DRD4	variable number of tandem repeats (VNTR)	7 repeats (longer: 7R+)	N/A	-	-	
Santoro *et al*, Am J Clin Nutr, 2007 [68]	*MC3R*	C17A (Thr6Lys; rs3746619)	A (C)	N/A	Rare allele carriers: ↓ $p = 0.03$	N/A	N/A
		G241A (Val81Ile; rs3827103)	A (G)	N/A	Rare allele carriers: ↓ $p < 0.05$	N/A	
Scherag *et al*, Obesity, 2011 [69]	*FTO*	rs1558902	A (T)	N/A	-	N/A	Additive
		rs9935401	A (G)		-		
	MC4R	rs17700144	A (G)		-		
	TMEM18	rs11127485	C (T)		-		

	TNKS-MSRA	rs17150703	A (G)		-		
		rs13278851	A (G)		-		
		rs516175	A (G)		-		
	SDCCAG8 (Examined both in children and adults)	rs10926984	T (G)		Children TT carriers: ↓ $P < 10^{-6}$ Adults: -		
		rs12145833	T (G)		Children TT carriers: ↓ $P < 10^{-6}$ Adults: -		
		rs2783963	C (T)		Children CC carriers: ↓		

					$P < 10^{-6}$<br>Adults: -		
Schum *et al*, Exp Clin Endocrinol Diabetes, 2012 [70]	*FTO*	rs1421085	C(T)	N/A	N/A	N/A	N/A
		rs17817449	G (T)				
		rs9939609	A (T)				
		rs1421085 + rs17817449 + rs9939609	Group 1: heterozygotes: for all 3 risk alleles<br>Group *2:* homozygote for at least one risk allele<br>Group 3: Homozygotes for non-risk alleles only	-	-	-	

Vogel *et al*, Obes Facts, 2011 [71]	*MC4R*	rs17782313	C (T)	N/A	All subjects: - Female risk allele carriers: ↑ p = 0.021	N/A	Additive
		rs12970134	A (G)		All subjects: - Female risk allele carriers: ↑ p = 0.009		
Volckmar *et al*, Exp Clin Endocrinol Diabetes, 2013 [72]	*SH2B1*	rs7498665	G (A)	N/A	-	N/A	Additive, Dominant, Recessive
	APOB48R	rs180743	G (C)		-		
Zlatohlavek *et al*,	*FTO*	rs17817449	G (T)	-	GG carriers compared to	-	N/A

Clinical Biochemistry, 2013 [73]					carriers of at least one T allele ↑ P=0.05		
	MC4R	rs17782313	C (T)	-	CC carriers compared carriers of at least one T allele ↑ P = 0.03	Total fat: CC carriers ↑ P=0.03	
	FTO + *MC4R*	rs17817449 + rs17782313	All possible allele combinations examined	*FTO* GG and/or *MCR4* CC carriers: ↑ P=0.01	*FTO* GG and/or *MCR4* CC carriers: ↑ P = 0.01	-	
Zlatohlavek *et al*,	*TMEM-18*	rs4854344	T (G)	-	-	-	N/A

Med Sci Monit, 2018 [74]							
	NYD-SP18	rs6971091	A (G)	-	-	-	

Abbreviations: N/A: not available, -: not statistically significant, ↑ : greater reduction, ↓ : lesser reduction, GRS: genetic risk score.
